# Fas-Independent T-Cell Apoptosis by Dendritic Cells Controls Autoimmune Arthritis in MRL/*lpr* Mice

**DOI:** 10.1371/journal.pone.0048798

**Published:** 2012-12-12

**Authors:** Takashi Izawa, Tomoyuki Kondo, Mie Kurosawa, Ritsuko Oura, Kazuma Matsumoto, Eiji Tanaka, Akiko Yamada, Rieko Arakaki, Yasusei Kudo, Yoshio Hayashi, Naozumi Ishimaru

**Affiliations:** 1 Department of Oral Molecular Pathology, Institute of Health Biosciences, The University of Tokushima Graduate School, Tokushima, Japan; 2 Department of Orthodontics and Dentofacial Orthopedics, Institute of Health Biosciences, The University of Tokushima Graduate School, Tokushima, Japan; Institut Jacques Monod, France

## Abstract

**Background:**

Although autoimmunity in MRL/*lpr* mice occurs due to a defect in Fas-mediated cell death of T cells, the role of Fas-independent apoptosis in pathogenesis has rarely been investigated. We have recently reported that receptor activator of nuclear factor (NF)-κB ligand (RANKL)-activated dendritic cells (DCs) play a key role in the pathogenesis of rheumatoid arthritis (RA) in MRL/*lpr* mice. We here attempted to establish a new therapeutic strategy with RANKL-activated DCs in RA by controlling apoptosis of peripheral T cells. Repeated transfer of RANKL-activated DCs into MRL/*lpr* mice was tested to determine whether this had a therapeutic effect on autoimmunity.

**Methods and Finding:**

Cellular and molecular mechanisms of Fas-independent apoptosis of T cells induced by the DCs were investigated by *in vitro* and *in vivo* analyses. We demonstrated that repeated transfers of RANKL-activated DCs into MRL/*lpr* mice resulted in therapeutic effects on RA lesions and lymphoproliferation due to declines of CD4^+^ T, B, and CD4^−^CD8^−^ double negative (DN) T cells. We also found that the Fas-independent T-cell apoptosis was induced by a direct interaction between tumor necrosis factor (TNF)-related apoptosis-inducing ligand-receptor 2 (TRAIL-R2) on T cells and TRAIL on Fas-deficient DCs in MRL/*lpr* mice.

**Conclusion:**

These results strongly suggest that a novel Fas-independent apoptosis pathway in T cells maintains peripheral tolerance and thus controls autoimmunity in MRL/*lpr* mice.

## Introduction

Rheumatoid arthritis (RA) is an autoimmune disease characterized by chronic inflammation and synovial infiltration of immune cells [1]. Various immune cells are implicated in the pathogenesis of RA in patients and in murine models [2]. Furthermore, interactions between osteoclasts and immune cells, such as T-cell priming by activated dendritic cells (DCs), may contribute to the pathogenesis of RA in human and murine models [3].

DCs are professional antigen-presenting cells (APCs) that are present in low numbers in all body tissues [4]. Immature DCs are capable of antigen uptake. After activation via Toll like receptor triggering [5], [6], RANK/RANKL [7], or CD40/CD40L signaling [8], [9], DCs are activated as evidenced by an up-regulation of MHC molecules and costimulatory molecules, such as CD40, CD80, and CD86 [10]. These mature DCs are no longer capable of antigen uptake but are endowed with the capacity to initiate antigen-specific T-cell responses. In contrast, immature DCs are believed to induce antigen-specific tolerance via the induction of regulatory T cells or the deletion of antigen-specific T cells [11]. Thus, DCs play a pivotal role in orchestrating the immune response against self and non-self antigens. Although several studies have demonstrated that DCs control autoimmunity in several diseases, including in RA [12], [13], it remains unclear how DCs regulate autoreactive T cells in the periphery.

We recently reported that crosstalk between Fas and receptor activator of NF-κB ligand (RANKL) maintains peripheral DCs associated with autoimmunity [14]. RANKL, a type II membrane protein of tumor necrosis factor (TNF) family, is expressed on osteoblasts, stromal cells, and activated T cells, and binds to the signaling receptor RANK and decoy receptor osteoprotegerin [7], [15]–[18]. RANK is widely expressed in the myelomonocytic lineage, ranging from osteoclast precursors to mature DCs [15], [19]. Mice lacking RANKL or RANK display severely reduced osteoclastogenesis, show defects in early differentiation of T and B cells, lack lymph nodes (LNs), and fail to develop mammary glands [20], [21]. Although we demonstrated that activation of Fas-deficient DCs was up-regulated by engagement of RANKL signaling, and that the single transfer of RANKL-stimulated DCs resulted in accelerated autoimmune arthritis in MRL/*lpr* mice [14], we speculated whether repeated transfers, but not single transfer, of RANKL-stimulated DCs modify peripheral tolerance and control autoimmunity in MRL/*lpr* mice.

In this study, we investigated the precise molecular mechanism of the interaction between activated DCs and T cells in the autoimmune response of MRL/*lpr* mice. Furthermore, a proposed new DC therapy was tested to see if it would regulate RA lesions in MRL/*lpr* mice.

## Results

### Therapeutic effect of repeated transfers of DCs on RA lesions in MRL/*lpr* mice

**Figure 1:**
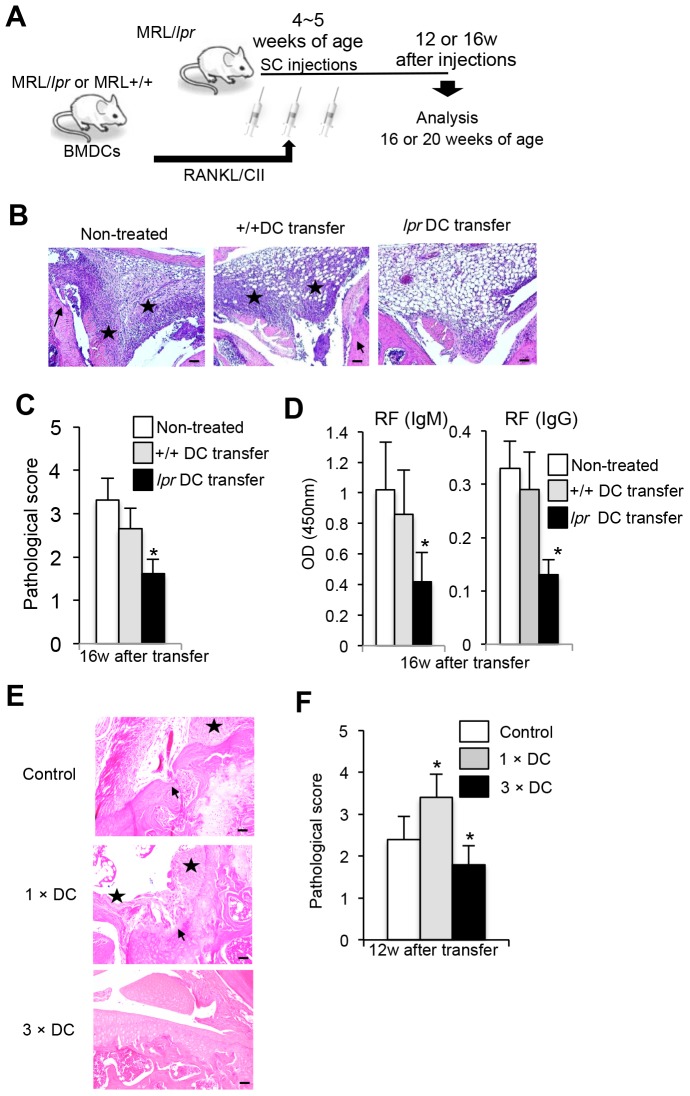
Therapeutic effect of repeated transfers of DCs on autoimmune arthritis. (A) Experimental protocol is shown. BMDCs from female MRL+/+ and MRL/*lpr* mice were stimulated with RANKL and CII, and then female MRL/*lpr* mice received a total of 3 injections of the BMDCs every other day distributed over 6 day period. At 16 weeks after transfer (20 weeks of age), the recipient MRL/*lpr* mice were analyzed. (B) Histology of joint from recipient mice. Histological photos with HE staining are shown as representative of the recipient mice at 16 weeks after transfers. Arrow; bone erosion or synovial proliferation, star; mononuclear inflammatory infiltrate, fibrosis, or panus. Scale bar: 100 µm (n = 7, 10 and 12 per group respectively). (C) The histological score of the recipient mice was evaluated at 16 weeks after repeated transfers. Data are shown as means ± SD. (n = 7, 10 and 12 per group respectively). (D) Rheumatoid factor (RF) (IgM and IgG) antibody was measured by ELISA. Values are shown as means ± SD (n = 7, 10 and 12 per group respectively). OD = optical density. (E) RA lesions of control, a single DC transferred (1× DC), and multiple DC transferred (3× DC) MRL/*lpr* mice were compared. Histological photos with HE staining are shown as representative of the recipient mice at 12 weeks after transfers. Scale bar: 100 µm (n = 5 per group respectively). (F) The histological score of the recipient mice was evaluated at 12 weeks after repeated transfers. Data are shown as means ± SD (n = 5 per group respectively). *p<0.05.

We have previously demonstrated that a single injection of RANKL and type II collagen (CII)-stimulated bone marrow-derived dendritic cells (BMDCs) into MRL/*lpr* mice resulted in elevated severity of RA lesions through up-regulation of T-cell functions including T-helper (Th)1-typed cytokine production or proliferative response [14]. We have also reported that the phenotype of the increased DC from MRL/*lpr* mice was myeloid DC showing CD11b^+^ CD11c^+^ CD8α^−^ [14]. Therefore, we hypothesized that multiple interactions of activated DCs with peripheral T cells can control autoimmunity. Thus we tried to analyze the regulatory mechanism of autoimmunity in MRL/*lpr* mice by multiple transfers of activated DCs. To elucidate how activated DCs regulate autoreactive T cells in the periphery, we performed repeated transfer experiments with RANKL and CII-activated DCs into MRL/*lpr* mice. As shown in Figure 1A, BMDCs from MRL/*lpr* or MRL/+/+ mice were stimulated with RANKL and CII, and subcutaneously transferred into MRL/*lpr* mice three times during a week from 4 to 5 weeks of age. At 16 weeks after the transfers, all the organs of the recipient MRL/*lpr* mice were analyzed. Pathological findings of RA lesions in non-treated MRL/*lpr* mice (20 weeks of age) showed subsynovial mononuclear inflammatory infiltrate, erosion and destruction of articular cartilage by panus, fibrosis, and synovial proliferation (Figure 1 B). Histological analysis showed that RA lesions from RANKL and CII-stimulated MRL/*lpr* DC- (*lpr* DC-) transferred mice were clearly improved although a slight infiltration of mononuclear cells was observed in the subsynovial connective tissue of the treated mice (Figure 1B). In contrast, there was not a significant effect of +/+ DC transfer on the RA lesion compared with that of *lpr* DC-transferred mice (Figure 1B). Histological evaluation revealed that the arthritic score of lesions from *lpr* DC-transferred mice was significantly lower than that from the control mice (Figure 1C). In addition, we compared RA lesions between RANKL-stimulated DCs- and RANKL+CII-stimulated DCs-transferred recipients. There was more therapeutic effect on RA lesions by multiple transfers of RANKL+CII-stimulated DCs than that of RANKL-stimulated DCs (Figure S1A). Furthermore, the levels of rheumatoid factor (RF) (IgM and IgG) in the sera of *lpr* DC-transferred mice were significantly reduced compared with those from controls (Figure 1D). Anti-double strand (ds)DNA and anti-CII Abs, but not anti-nuclear antibody (ANA), as well as RF in the recipients transferred with activated *lpr* DCs were significantly reduced compared with those in the recipients transferred with control DCs (Figure S1B). It is still unclear whether antibody against CII influences the induction of RA lesions in MRL/*lpr* mice. It has been reported that severe RA lesions can develop without anti-CII antidoby [22], [23]. However, it is possible that CII-primed DCs enhance *in vivo* immune reaction including CII-specific response in MRL/*lpr* mice. On the other hand, when *lpr* DCs stimulated without CII antigen were transferred into *lpr* recipients, autoantidoby production of the sera from the recipients was not changed (Figure S1C). Therefore, the antigen-specific response plays a key role in triggering the immunoregulatory mechanism in the recipient mice. When we compared a single transfer and multiple transfers (three times) of activated *lpr* DCs into MRL/*lpr* mice, there was a clear difference for severity of autoimmune lesions between these two treatments (Figure 1E and 1F). These results showed that repeated transfers of activated DCs could control RA lesions in MRL/*lpr* mice. In particular, *lpr* DCs activated with both RANKL and CII could regulate the RA lesion effectively.

### Effect of repeated transfers of activated DCs on lymphoproliferation in MRL/*lpr* mice

**Figure 2:**
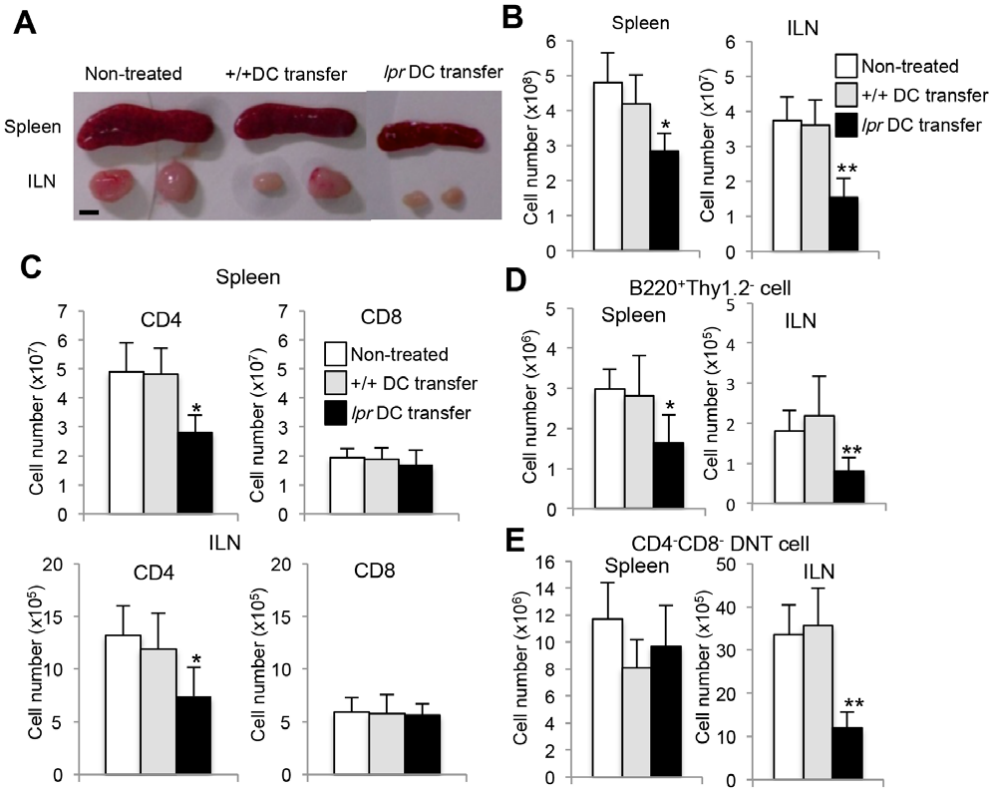
Reduced lymphoproliferation of MRL/*lpr* mice following repeated transfers of DCs. (A) Spleen and ILNs from the recipient mice are shown. Photos are representative of the recipient mice (16 weeks of age) at 12 weeks after the transfer. Values are shown as means ± SD (n = 5, 7 and 7 per group respectively). (B) The total cell number in the spleen, and ILNs is shown. Scale bar: 5 mm. (C) T cell numbers in the spleen and ILNs of the recipient mice. Flow cytometry was performed using spleen and ILN cells. The number of CD4^+^ and CD8^+^ T cells is shown. (D) B cell (B220^+^ Thy1.2^−^) number in the spleen and ILNs of the recipient mice. (E) CD4^−^ CD8^−^ CD3^+^ DNT cell number in the spleen and ILNs of the recipient mice. Values are shown as means ± SD (n = 5, 7, and 7 respectively per group). *p<0.05, **p<0.005.

It is well known that splenomegaly and systemic lymphoadenopathy are observed in MRL/*lpr* mice [24]–[26]. The size of the spleen and inguinal lymph nodes (ILNs) from *lpr* DC-transferred mice was smaller than those from control mice (Figure 2A). The total cell number of spleen and ILNs in *lpr* DC-transferred mice was also significantly decreased compared with that of control mice (Figure 2B). Furthermore, to clarify which subset of lymphocytes was reduced in the spleen and ILNs from *lpr* DC-transferred mice, the T cell subpopulation was analyzed by flow cytometry. The number of CD4^+^ T cells from the spleen and ILNs of *lpr* DC-transferred mice was significantly decreased compared with that of control mice (Figure 2C). In contrast, no difference was observed in the number of CD8^+^ T cells of the spleen and ILNs between *lpr* DC-transferred mice and control mice (Figure 2C). Moreover, the number of B220^+^Thy1.2^−^ B cells of spleen and ILNs from *lpr* DC-transferred mice was significantly reduced compared with that from control mice (Figure 2D). In addition, a significantly decreased number of CD4^−^CD8^−^ double negative (DN) T cells of ILNs, not spleen, in RANKL+CII-*lpr* DC-transferred mice was found (Figure 2E). Next we attempted to determine the T and B cell apoptosis and maturation *in vivo*. As we could not detect apoptosis of the cells at 8 or 12 weeks after the transfer, we analyzed apoptosis of T and B cells at 2 weeks after the transfer. Flow cytometric analysis showed that annexin-V^+^ CD4^+^ T, B, and DNT cells of ILNs from *lpr* DCs-transferred recipients were significantly increased compared with those from +/+ DCs-transferred recipients (Figure S2A, B, C, D). In addition, there were no differences in the frequency of memory (CD44^high^ CD62L^−^) CD4^+^ T cells between *lpr* and +/+ DCs-transferred recipients although CD44^high^ CD62L^+^ activated CD4^+^ T cells of *lpr* DC-transferred mice were relatively increased compared with that of controls (Figure S3A). As to B cell maturation markers (CD27 and CD5), there were no differences between three groups (Figure S3B). Those findings suggest that repeated interactions between Fas-deficient DCs and T cells regulate CD4^+^ T-cell activation. Additionally, the repeated transfers of DCs controlled B and CD4^−^ CD8^−^ DNT cell survival in the periphery and reduced lymphoproliferation as well as RA lesions in MRL/*lpr* mice.

On the other hand, when carboxyfluorescein succinimidyl ester (CFSE)-labeled +/+ or *lpr* DCs were subcutaneously injected into MRL/*lpr* mice, significantly increased CFSE^+^ CD11c^+^ *lpr* DCs were observed compared with those from MRL+/+ mice in ILNs at 2 weeks after the transfer (Figure S4A). Therefore, *in vivo* experiment shows that the survival of *lpr* DCs may be better than that of +/+ DCs. Moreover, we detected increased CFSE^+^CD11c^+^ cells in spleen as well as ILNs from *lpr* DC-transferred mice comparing with +/+ DC-transferred mice (Figure S4B). It is possible that a therapy using normal DCs may be effective for RA lesions by any manipulation for longer survival.

### T-cell functions in DC-transferred mice

**Figure 3:**
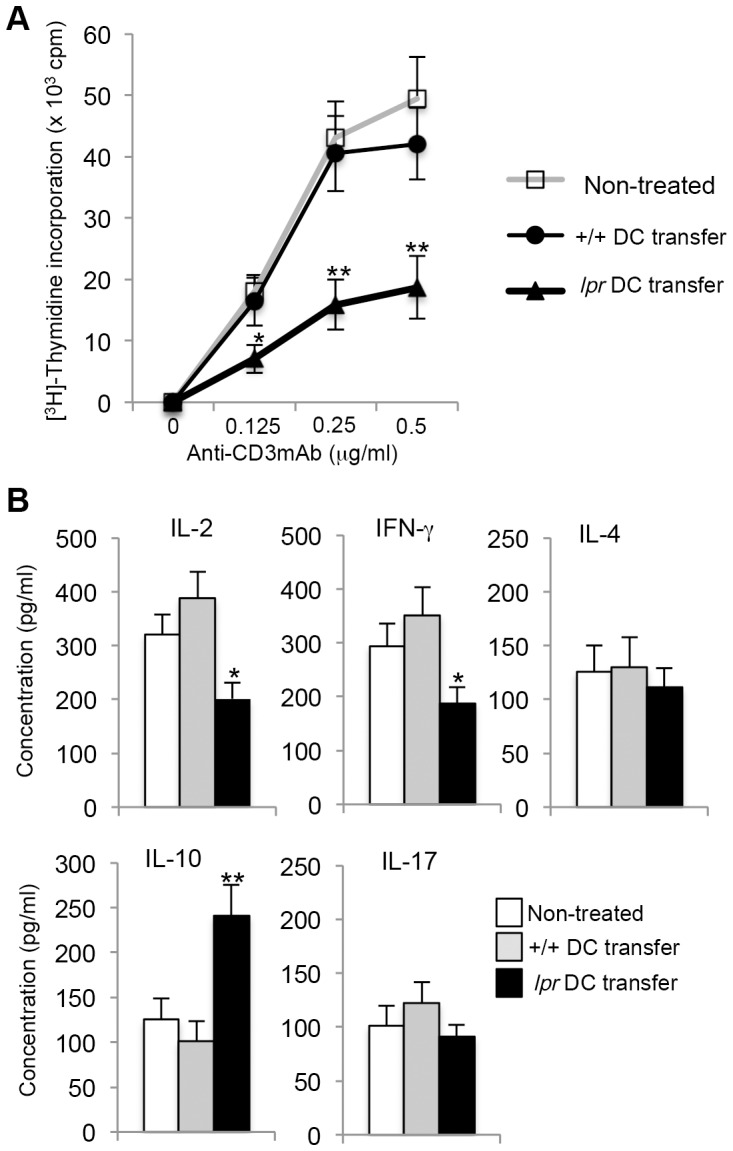
T cell responses in DC-transferred MRL/*lpr* mice. (A) Proliferative responses of ILN CD4^+^ T cells from the recipients and control mice were analyzed. Purified CD4^+^ T cells were stimulated with plate-coated CD3 mAb (0–0.5 µg/ml) and CD28 mAb (10 µg/ml) for 72 hours. The proliferative response was evaluated by [^3^H] thymidine incorporation. Values are means ± SD (n = 4, 5, and 5 respectively per group). Results are representative of three independent experiments with similar results. (B) The culture supernatants for 24 h (anti-CD3 mAb: 0.5 µg/ml; anti-CD28 mAb: 10 µg/ml) as described above were analyzed for cytokine productions including IL-2, IFN-γ, IL-4, IL-10, and IL-17 by ELISA. Values are means ± SD (n = 4, 5, and 5 respectively per group). *p<0.05, **p<0.005.

We next evaluated T-cell functions of *lpr* DC-transferred mice at 12 weeks after the transfer. Purified CD4^+^ T cells from ILNs of recipient MRL/*lpr* mice were stimulated with plate-coated anti-CD3 monoclonal antibody (mAb) (0–0.5 µg/ml) and-CD28 mAb (10 µg/ml) for 72 hours to analyze proliferation with the incorporation of [^3^H]-Thymidine. T-cell response in ILNs from *lpr* DC-transferred MRL/*lpr* mice was significantly decreased compared with that form +/+ DC-transferred and control mice (Figure 3A). By contrast, when T cells from the recipient MRL+/+ mice transferred with multiple transfers of activated DCs were analyzed, there was no change in the proliferation of CD4^+^ T cells between three groups (Figure S5). Moreover, cytokine productions using the culture supernatants from anti-CD3 mAb-engaged CD4^+^ T cells of spleen and ILNs were analyzed by ELISA. Th1-typed cytokine production such as IL-2 and IFN-γ from *lpr* DC-transferred MRL/*lpr* mice was significantly lower than that from +/+ DC-transferred and control mice (Figure 3B). By contrast, IL-10 production in the ILN CD4^+^ T cells from *lpr* DC-transferred MRL/*lpr* mice was significantly enhanced compared with that from +/+ DC-transferred recipients (Figure 3B). By repeated transfer of the DCs, the immune environment displaying Th1 cytokine profile of CD4^+^ T cells was shifted to Th2 cytokine profile including IL-10. It was possible that the induction of IL-10-dependent tolerogenic environment by multiple DC transfers might play a crucial role in the progression of autoimmunity in MRL/*lpr* mice. As for IL-4 and IL-17 production, there was no significant difference between *lpr* DC-transferred and control mice (Figure 3B). These results indicate that activated DCs crucially regulate the peripheral T-cell functions in MRL/*lpr* mice. Activated and CII-exposed *lpr* DC may be capable of controlling T-cell survival in the periphery by continuing the stimulation. As for signal II initiated by CD28 ligation on T cells, the results of T-cell functions suggest that the T cell signaling controlled by signal I, II, and III may be imbalanced in the DC-transferred recipient mice. Therefore, if normal DCs can survive to continue stimulating T cells like activated *lpr* DCs, it is possible that normal DCs might induce the same effect with the imbalance of T cell signaling. In addition, we performed the flow cytometric analysis of thymic T cells (CD4 and CD8) of the treated recipient mice (Figure S6A). There was no change between the treated and control mice. Therefore, multiple transfers of DCs could not influence T cell differentiation in the thymus. Moreover, we analyzed regulatory T (T_reg_) cells of ILNs and spleen in the recipient MRL/*lpr* mice treated with multiple transfers of DCs. There was no difference in the frequency of CD25^+^ Foxp3^+^ CD4^+^ T_reg_ cell of ILNs and spleen between +/+ DCs- and *lpr* DCs-transferred recipients (Figure S6B).

### T-cell apoptosis induced by activated DCs

**Figure 4:**
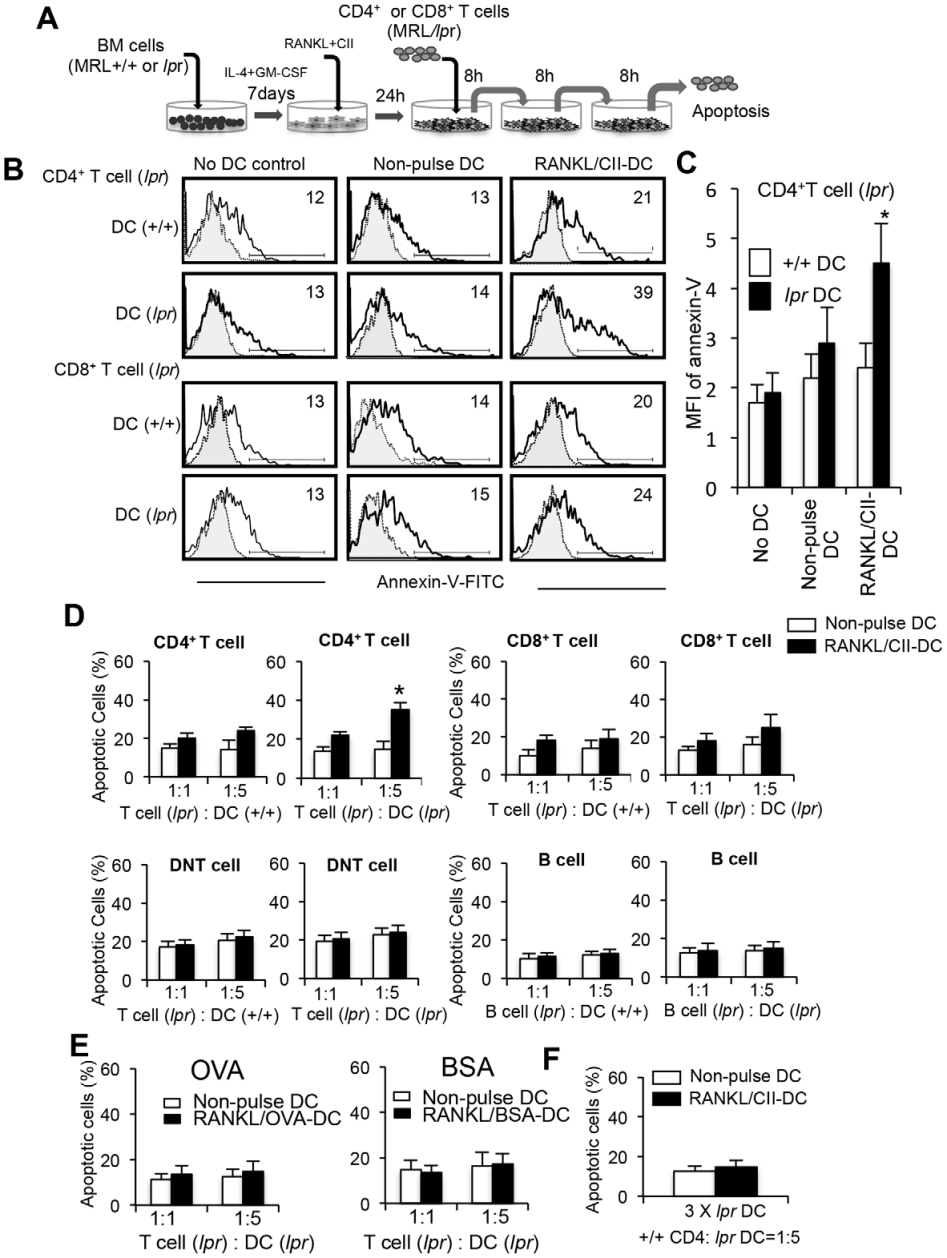
Fas-independent T-cell apoptosis in DC-transferred MRL/*lpr* mice. (A) Experimental protocol of T cell apoptosis by repeated co-culture with DCs. Total T cells from MRL/*lpr* mice (5×10^4^) were repeatedly (three times) co-cultured with BMDCs (2.5×10^5^) from MRL+/+ or MRL/*lpr* mice for 8 hours without interval. BMDCs were stimulated with RANKL and CII for 24 hours before the co-culturing. (B) After the third co-culture, apoptosis of CD4^+^ and CD8^+^ T cells expressing annexin-V was detected by flow cytometry. Staining of T cell with FITC-labeled isotype control Ab is shown as a dotted line. Results are shown as representative of three independent experiments with similar results. (C) MFI of annexin-V on CD4^+^ T cells was calculated, and the data are shown as the means ± SD of triplicate samples. (D) Induction of T-cell apoptosis by repeated co-culturing with activated DCs. Purified CD4^+^, CD8^+^, DNT, and B220^+^ cells (5×10^4^) were repeatedly co-cultured with BMDCs (5 and 25×10^4^). Annexin-V^+^ cells are shown as the means ± SD of triplicate samples. The experiments were repeated three times with similar results. *p<0.05. (E) BMDCs were stimulated with RANKL and, OVA or BSA (10 µg/ml) for 24 hours before the co-culturing. Purified CD4^+^ cells (5×10^4^) were repeatedly co-cultured with BMDCs (5 and 25×10^4^). Annexin-V^+^ cells are shown as the means ± SD of triplicate samples. The experiments were repeated three times with similar results. (F) Purified CD4^+^ cells (5×10^4^) from ILNs in MRL+/+ mice were repeatedly co-cultured with *lpr* BMDCs (25×10^4^). Annexin-V^+^ cells are shown as the means ± SD of triplicate samples. The experiments were repeated three times with similar results.

Repeated DC transfers reduced the cell number of CD4^+^ T cells in MRL/*lpr* mice. However, it remained unclear whether apoptosis of CD4^+^ T cells could be induced by repeated interactions with DCs. Thus, T cells of ILNs from MRL/*lpr* mice were repeatedly (three times) co-cultured with RANKL and CII-stimulated BMDCs from MRL/*lpr* or MRL+/+ mice (Figure 4A). Although the *in vivo* immune response in the recipient treated with multiple transfer was not clear, *in vitro* repeated interactions of activated DCs with T cells could be one of clues to understand the *in vivo* immune response. In brief, T cells were repeatedly transferred into each well in which T cells were co-cultured with RANKL and CII-stimulated-*lpr* or +/+ DCs for 24 hours. After the third incubation, apoptotic cells expressing annexin-V were detected by flow cytometry. Apoptosis of CD4^+^, but not CD8^+^, T cells co-cultured with *lpr* DCs was significantly increased compared with those incubated with +/+ DCs (Figure 4B). In addition, when compared the mean fluorescence intensity (MFI) of annexin-V on the co-cultured CD4^+^ T cells, the MFI on CD4^+^ T cells co-cultured with RANKL and CII-stimulated *lpr* DCs was significantly increased in contrast to that with RANKL and CII-stimulated +/+ DCs (Figure 4C). The CD4^+^ T-cell apoptosis was induced by *lpr* DCs dependent on the number of DCs (Figure 4B). In contrast, apoptosis of CD8^+^ T cells was not enhanced by repeated co-culturing with *lpr* DCs (Figure 4B). There was no increased apoptosis of DNT cells *in vitro* by repeated interactions with DCs (Figure 4D). In addition, apoptosis of B220^+^ Thy1.2^−^ B cells from MRL/*lpr* and MRL+/+ mice was not induced by the repeated co-culture with *lpr* DCs (Figure 4D). We confirmed that the number of living and dead cells before the co-culture with T cells was not changed after the co-culture for 24 hours. While it is possible that *lpr* CD4^+^ T cells may control DNT cells and B cells in the periphery directly or indirectly, there may be still veiled *in vivo* mechanism of the survival of abnormal DNT cells. As for the cultured BMDCs, we prepared the same number between +/+ and *lpr* DCs (Figure 4). When ovalbumin (OVA) or bovine serum albumin (BSA), and RANKL-stimulated *lpr* DCs were repeatedly co-cultured with CD4^+^ T cells from MRL/*lpr* mice, there was no significant increase of apoptotic cells in contrast to the co-culture with CII and RANKL-stimulated *lpr* DCs (Figure 4E). Additionally, we performed the *in vitro* experiment using CD4^+^ T cells from MRL+/+ mice. When CD4^+^ T cells from MRL+/+ mice were co-cultured repeatedly with RANKL+CII-stimulated *lpr* DCs, a significant increase of +/+ CD4^+^ T cell apoptosis like *lpr* CD4^+^ T cells was not observed (Figure 4F). These results suggest that Fas-independent T-cell apoptosis is induced by repeated interactions of activated DCs. However, the precise mechanism of *in vivo* immune response in the recipient treated with multiple transfer has not been clear.

### Molecular mechanism of Fas-independent T-cell apoptosis

**Figure 5:**
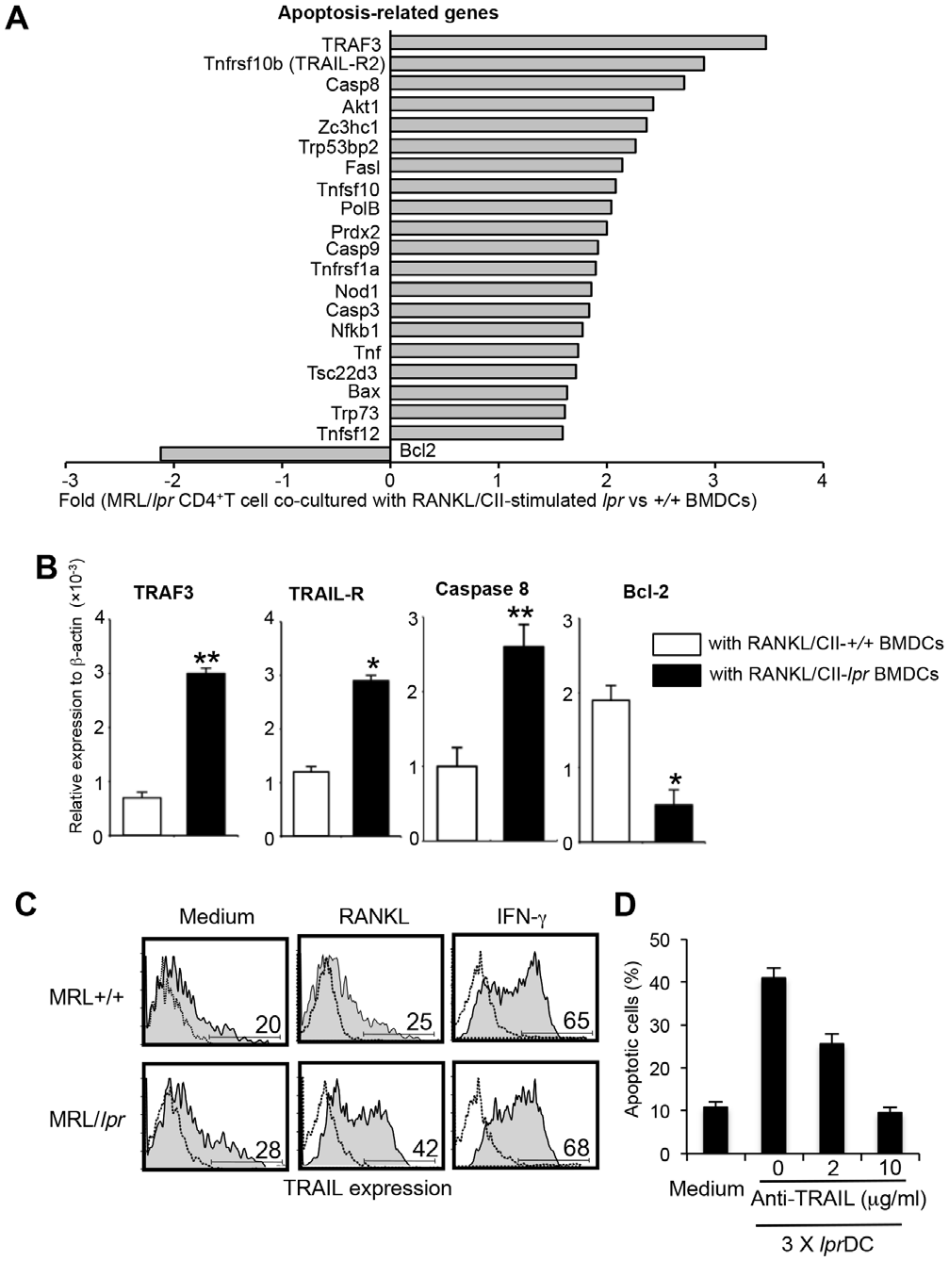
T-cell apoptosis via TRAIL/TRAIL-R2. (A) Real-time RT-PCR for a wide array of apoptosis-related genes was performed using mRNA samples of MRL/*lpr* CD4^+^ T cells repeatedly stimulated with MRL/*lpr* and MRL+/+ BMDCs. Gene expression of CD4^+^ T cells repeatedly stimulated with MRL/*lpr* BMDCs was compared with those stimulated with MRL+/+ BMDCs (controls). Genes with increased and decreased expression are shown as fold of control. The experiments were repeated twice with similar results. (B) The mRNA expression of TRAF3, TRAIL-R2, caspase 8, and Bcl-2 was confirmed by quantitative real-time PCR analysis. Relative expression to β-actin level is shown as means ± SD from triplicate samples. The experiments were repeated three times with similar results. (C) Up-regulation of TRAIL expression on MRL/*lpr* BMDCs by RANKL was detected by flow cytometry. Staining of DC with FITC-labeled isotype control Ab is shown as a dotted line. The experiments were repeated three times with similar results. (D) *lpr* CD4+ T cells were repeatedly co-cultured with activated *lpr* DCs in the presence of anti-TRAIL mAb. Data are shown as means ± SD of triplicate samples. *p<0.05, **p<0.005.

To elucidate the molecular mechanism responsible for Fas-independent T-cell apoptosis, we compared the gene expression of RANKL/CII-*lpr* DC-stimulated *lpr* CD4^+^ T cells with RANKL/CII-*+/+* DC-stimulated *lpr* CD4^+^ T cells using a PCR-based SuperArray method focusing on apoptosis-related genes. Of the 96 genes analyzed, the most increased gene was TRAF3 (>3-fold), and 10 genes including TNFSF10b (TRAIL-R2), and caspase 8 showed >2-fold increase compared with *+/+* DC-stimulated CD4^+^ T cells (Figure 5A). It has been reported that TRAIL-R plays an important role in activation-induced apoptosis of CD4^+^ T cells [27]. Therefore, we hypothesized that Fas-independent apoptosis of CD4^+^ T cells is induced by the interaction between TRAIL-R2 on CD4^+^ T cells and TRAIL on activated DCs in MRL/*lpr* mice. To confirm the result of the PCR-array, mRNAs of the up-regulated genes were evaluated by quantitative RT-PCR. Consistent with the data from the RCR-array, mRNAs of TRAF3, TRAIL-R2, and caspase 8 of T cells stimulated with *lpr* DCs were significantly increased compared with those from control T cells (Figure 5B). In contrast, the anti-apoptotic gene Bcl-2 was significantly decreased (Figure 5B). Next, we examined TRAIL expression on RANKL-stimulated DCs from MRL/*lpr* mice. Although a previous report demonstrated that TRAIL expression on DCs was up-regulated by IFN-γ stimulation [28], it was unclear whether TRAIL on DCs can be controlled by the RANK/RANKL signal. In our study, TRAIL expression on DCs from MRL/*lpr* mice was significantly enhanced by RANKL stimulation (Figure 5C). No difference was observed in the increased expression of *lpr*DCs and +/+ DCs induced by IFN-γ stimulation (Figure 5C). Moreover, we performed an additional experiment using anti-TRAIL mAb to block *in vitro* T cell apoptosis by multiple interactions with activated DCs. An anti-TRAIL mAb could inhibit *in vitro lpr* T-cell apoptosis by the interactions with activated *lpr* DCs (Figure 5D). These results suggest that Fas-independent T-cell apoptosis is induced by a direct interaction between TRAIL-R on T cells and TRAIL on DCs. This shows that apoptosis of Fas-deficient CD4^+^ T cells may be controlled through TRAIL/TRAIL-R. Therefore, although normal T cells are resistant to TRAIL/TRAIL-R-mediated apoptosis as described in the previous report [29], the maintenance of peripheral T cells in human patients with abnormal Fas/FasL system may be regulated by the TRAIL/TRAIL-R-mediated pathway.

### TRAIL/TRAIL-R2-mediated apoptosis of Fas-deficient CD4^+^ T cells

**Figure 6:**
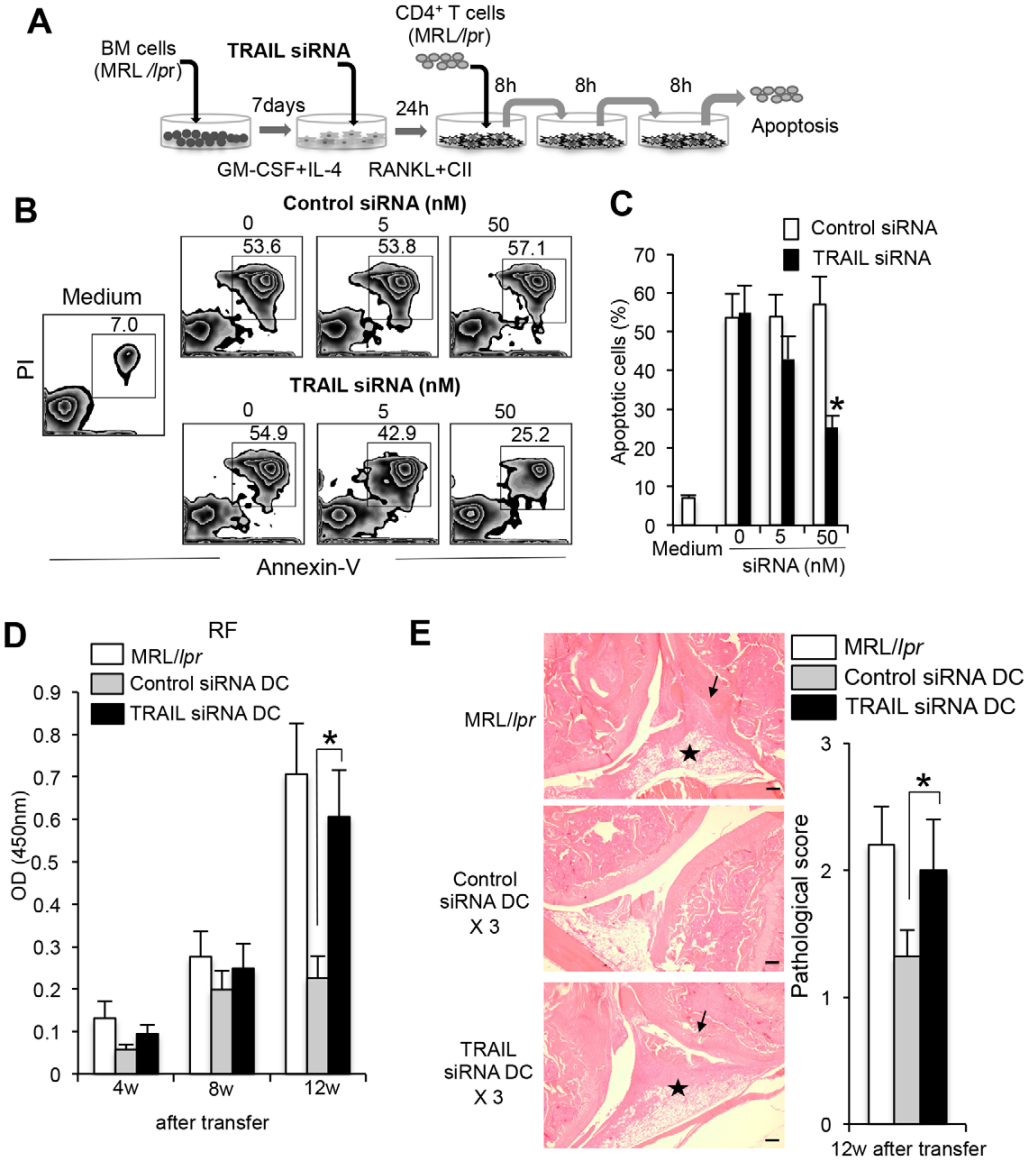
Regulation of Fas-independent T cell apoptosis by TRAIL siRNA-treated DCs. (A) BMDCs from MRL/*lpr* mice were treated with TRAIL gene-specific siRNA or control siRNA for 24 hours, and then stimulated with RANKL and CII for 48 hours. Purified CD4^+^ T cells of LNs from MRL/*lpr* mice were repeatedly (three times) co-cultured with the activated DCs for 8 hours by transfer into each new well. (B) Expression of TRAIL on activated BMDCs treated with TRAIL siRNA or control siRNA was analyzed by flow cytometry. Results are representative of two independent experiments with similar results. (C) Apoptosis of CD4^+^ T cells cocultured with siRNA-treated DCs was analyzed by flow cytometry with Annexin-V and PI. Results are representative of two independent experiments. (D) Apoptotic cells (%) are shown as the mean ± SD from triplicate samples. The experiments were repeated three times with similar results. (E) *In vitro* TRAIL siRNA-treated and control DCs were injected three times into MRL/*lpr* mice (4 weeks of age). After 12 weeks after the transfers, RF level of sera from the recipient mice (16 weeks of age) was detected by ELISA. Values are means ± SD. (n = 5). The experiments were repeated twice with similar results. (F) Histology of joint from recipient mice. Histological photos with HE staining are shown as representative of five mice in each group at 12 weeks after transfers. Arrow; bone erosion or synovial proliferation, star; mononuclear inflammatory infiltrate, fibrosis, or panus. Scale bar: 100 µm. Histological score is shown as means ± SD. (n = 5) *p<0.05.

To further confirm TRAIL/TRAIL-R2-mediated apoptosis of Fas-deficient CD4^+^ T cells by RANKL+CII-stimulated DCs, we examined whether siRNA for TRAIL gene silencing inhibits T-cell apoptosis. BMDCs from MRL/*lpr* mice were treated with TRAIL gene-specific siRNA, and then stimulated with RANKL and CII for 48 hours. During the last 24 hours of the culture, purified CD4^+^ T cells from MRL/*lpr* mice were repeatedly (three times) co-cultured with the DCs for 8 hours. Apoptotic cells (annexin-V^+^PI^+^) were analyzed by flow cytometry as shown in Figure 6A. When the effect of the TRAIL gene-specific siRNA on the surface expression of DCs from MRL/*lpr* mice was evaluated, up-regulated TRAIL expression on RANKL+CII-stimulated DCs was seen to decrease in a dose-dependent manner, indicating that the knockdown was effective (Figure S7A, B). By contrast, the increased level of TRAIL in stimulated DCs was unchanged by treatment with control siRNA (Figure S7A, B). Interestingly, apoptosis of CD4^+^ T cells induced by repeated co-culturing with RANKL and CII-stimulated DCs was significantly reduced by treatment with TRAIL siRNA although there was no change in T-cell apoptosis following co-culturing with control siRNA-treated DCs (Figure 6B, C). Furthermore, we assessed the repeated transfer using TRAIL siRNA-treated DCs. BMDCs from MRL/*lpr* mice were treated with TRAIL siRNA *in vitro*, and then repeatedly transferred into MRL/*lpr* mice during 4 to 5 weeks of age. At 12 weeks (16 weeks of age) after the transfers, autoantibody production of serum in the recipients such as RF was measured by ELISA. Although serum titer of RF in the recipient MRL/*lpr* mice transferred with control siRNA-treated DCs were significantly decreased compared with untreated MRL/*lpr* mice at 4, 8, and 12 weeks after the transfers, RF titer of the MRL/*lpr* recipients transferred with TRAIL siRNA-treated DCs was not reduced, and was equal to that of control MRL/*lpr* mice (Figure 6D). In addition to RF, we analyzed anti-dsDNA and anti-CII Abs. We could detect a significant increase of anti-dsDNA and anti-CII Abs in the recipient transferred with TRAIL siRNA-treated DCs compared with that with control siRNA-treated DCs (Figure S8). Moreover, histological analysis showed that the therapeutic effect of repeated transfers of DCs on RA lesions was inhibited by *in vitro* treatment with TRAIL siRNA for DCs (Figure 6E). This result indicates that activated DCs expressing TRAIL plays a key role in regulating Fas-independent apoptosis of peripheral T cells.

## Discussion

DCs are crucial for the initiation of T-cell immunity and play an important role in the onset and regulation of immune responses in RA [13], [30]. Our previous report demonstrated that a single transfer of RANKL-stimulated DCs resulted in the exacerbation of RA lesions in MRL/*lpr* mice [14]. In contrast, the present study revealed the therapeutic effect of repeated transfers of DCs on the RA lesions and lymphoproliferation in MRL/*lpr* mice. In terms of recent therapeutic strategies for RA, modulation of several cytokines, such as TNF-α, IL-1, and IL-6 are therapeutic targets in RA [2], [31]. However, since cytokines regulate a broad range of inflammatory processes and since this regulatory network is considerably complicated in the pathogenesis of RA, the clinical application of such therapies is risky because of potential side effects on the immune system. In this study, no changes were observed in any other organs in recipient mice that were subjected to repeated transfers of DCs when all the organs were histopathologically examined. It has been reported that repeated injections of DCs matured with TNF-α induces antigen-specific protection against experimental autoimmune encephalomyelitis (EAE) in mice [32]. Although it was reported that overexpression of IL-10 is associated with the manifestations of ALPS and SLE, the reduced Th2 cell population producing IL-10 is related to the disease severity in RA [33]–[36]. In our study, since *lpr* DC therapy was effective for RA lesions, but not renal lesions, there might be different effects of DC transfer on autoimmune lesions in each target organ. Regarding antigen-specificity in our model, RANKL-stimulated DCs were incubated with CII antigen *in vitro* in this study and in our previous report. Without CII antigen incubation, the significant effects of DC transfer on autoimmunity were not fully observed. Although we have not clarified “antigen specificity” using only CII antigen, *in vitro* experiment using OVA or BSA antigen implies that CII antigen may play a important role in triggering the onset of autoimmunity of MRL/*lpr* mice. Therefore, DC therapy with CII antigen in addition to potent stimulation by RANKL can be more effective for autoimmunity.

We transferred the DCs into MRL/*lpr* at 4 weeks of age in which lymphadenopathy and splenomegaly based on lymphproliferation of the mice was not observed. By the multiple transfers of DCs, CD4^+^ T cells might be killed directly by *lpr* DCs while it is possible that apoptosis of DNT cell and B cells might be indirectly induced. According to our preliminary experiment, multiple transfers of DCs at 10 weeks of age could not be effective for suppression of RA lesion and lymphadenopathy of MRL/*lpr* mice. In this study, it is suggested that beneficial of the multiple transfers of activated DCs is confined to the *lpr* background and antigen-specific immune response.

Our previous report demonstrated that crosstalk between RANKL and Fas signaling in DCs controls autoimmune arthritis in MRL/*lpr* mice [14]. Moreover, it was reported that RANKL regulates Fas expression and Fas-mediated apoptosis in osteoclast [37]. To determine whether such control of autoimmunity by DCs could be used as a therapeutic strategy, repeated transfers of activated DCs were performed in this study to see if there could prevent autoimmune arthritis in MRL/*lpr* mice. We found that TRAIL expression on BMDCs from MRL/*lpr* mice was up-regulated by RANKL stimulation. TRAIL is known to interact with at least two death receptors, including death receptor 4 (DR4, TRAIL-R4) and death receptor 5 (DR5, TRAIL-R5), and two decoy receptors (decoy receptor 1 [DcR1, TRAIL-R3, TRID] and decoy receptor 2 [DcR2, TRAIL-R4, TRUNDD]) [38]–[40]. Apoptosis through TRAIL/TRAIL-R has been reported in several tumor cell lines [38]. The apoptosis is mediated by DR4 and DR5, which possess intracellular death domains similar to those of TNF receptor I and Fas [38], [41]. In addition, death domains of TRAIL-R activate mitochondria-dependent and mitochondria-independent pathways of apoptosis through FADD-caspase 8, leading to activation of the caspase cascade [42]–[44]. A previous report described that TRAIL-overexpressed DCs could inhibit the development of CII-induced arthritis (CIA) [45]. Although the precise molecular mechanism is obscure, it is possible that RANKL-induced TRAIL expression on DCs from MRL/*lpr* mice triggers T cell apoptosis. Abnormal system of Fas/FasL is not found in all other models and in all human RA. Autoimmunity is known to be caused by multi-factors, and is a complex disease. RA lesions in MRL/*lpr* mice resembling human RA are the most common among RA animal models. Therefore, the abnormality of Fas/FasL system in immune cells is considered to influence the pathogenesis of human RA. Fas-deficient DCs might be more useful than normal DCs expressing Fas molecule for treatment of autoimmunity in our study. The fact that Fas-deficient DCs become more activated than normal DCs could affect Fas-independent T cell apoptosis to prevent autoimmunity in MRL/*lpr* mice. This suggests that DC therapy might be helpful for autoimmunity of patients with abnormal Fas expression on the immune cells, and that a therapy for autoimmunity using normal DCs might fail to prevent or treat autoimmune diseases. Although it is still difficult to apply a new DC therapy for human RA, any therapeutic strategy with controlling autoreactive T cells by DCs will be useful in the feature. In addition, although the effect observed in our study is confined to MRL/*lpr* mice, the mice are the most common and useful for understanding the pathogenesis of autoimmune RA. Therefore, any unique phenomenon or effect using MRL/*lpr* mice will pave to a road to define the mechanism of autoimmunity and develop any new therapy for autoimmunity.

Cell death of peripheral T cells is one of the systems used to maintain immunological tolerance [46]. Fas/FasL in T cell apoptosis plays a crucial role in the maintenance of peripheral tolerance [46]. Although the relationship between apoptosis of peripheral T cells and TRAIL/TRAIL-R2 is unclear, it has been reported that mice deficient in TRAIL have a severe defect in thymocyte apoptosis and that TRAIL is important in the induction of autoimmune diseases [47], [48]. TRAIL/TRAIL-R-mediated T-cell apoptosis may be promoted in a Fas-deficient situation by an interaction with DCs that highly express TRAIL. In contrast, it has also been reported that reciprocal expression of TRAIL and FasL in T helper 1 and 2 cells plays a key role in T-cell apoptosis in T helper subset differentiation [27]. Our results suggest that activated Fas-deficient T cells expressing TRAIL-R is induced by repeated interactions with TRAIL-expressing DCs. It is possible that TRAIL/TRAIL-R-mediated apoptosis of T cells plays a key role in an alternative of apoptosis pathway.

In summary, repeated transfers of activated Fas-deficient DCs resulted in a therapeutic effect on lymphoproliferation and autoimmune arthritis in MRL/*lpr* mice due to Fas-independent apoptosis of CD4^+^ T cells through TRAIL/TRAIL-R2. Our new therapeutic approach using this alternative apoptosis pathway could prove to be a powerful strategy for the prevention and cure of immune disorders in the near future.

## Materials and Methods

### Ethics

This study was conducted according the principles expressed in the Declaration of Helsinki. The study was approved by the Institutional Review Board of the University of Tokushima (toku09021).

### Mice

MRL/*Mp-lpr*/*lpr* mice (MRL/*lpr*: aged 4–12 weeks; n = 75) and MRL*+/+* mice (aged 4–12 weeks; n = 50) were purchased from Japan SLC (Shizuoka, Japan). All mice were maintained under specific pathogen-free conditions at our animal facility. We analyzed female mice at 16 or 20 weeks of age.

### Bone marrow-derived DCs (BMDCs)

BMDCs were generated from the bone marrow of MRL/*lpr* or MRL*+/+* mice as described previously [49]. BMDCs were stimulated with 100 ng/mL RANKL and 50 µg/mL chicken type II collagen (CII) for 48 hours. 2×10^6^ BMDCs/a mouse were transferred three times or once into recipient MRL/*lpr* mice at the base of the tail by subcutaneous injections in 200 µL PBS at the age of 4 weeks.

### Histopathology

All organs were taken from the mice, fixed with 4% phosphate-buffered formaldehyde (pH 7.2), and prepared for histological examination. The sections (4 µm in thickness) were stained with hematoxylin and eosin (HE). Histological grading of inflammatory arthritis was performed according to the methods of Edwards *et al* [42]. as follows: a 1-point score indicates hyperplasia/hypertrophy of synovial cells, fibrosis/fibroplasia, proliferation of cartilage and bone, destruction of cartilage and bone, and mononuclear cell infiltrate. We have confirmed that any inflammatory findings are observed in over 90% MRL/*lpr* mice at 16 or 20 weeks of age. In approximately 20% female MRL/*lpr* mice at 12 weeks of age, any slight findings such as hyperplasia of synovial cells and mononuclear cell infiltration were observed. These findings of arthritis lesions in MRL/*lpr* mice are consistent with those in previously demonstrated reports [50]–[53].

### Flow cytometry

Spleen and LN cells were stained with fluorescein isothiocyanate (FITC)-conjugated anti-CD8, phycoerythrin (PE)-conjugated anti-CD4, PE-Cy5.5-conjugated anti-CD44, APC-conjugated anti-CD62L, FITC-conjugated anti-CD5, APC-conjugated anti-CD27, PE-conjugated anti-B220, PE-Cy7-conjugated anti-CD19-C, PE-conjugated anti-TRAIL mAb (eBiosciences, San Diego, CA, USA). Cells were analyzed by a FACScan (BD Biosciences, Franklin Lakes, NJ, USA).

### Proliferation assay

T cells (>90%) were enriched from single-cell suspensions of spleen and ILN cells from recipient MRL/*lpr* mice with nylon wool (Wako Pure Chemical, Tokyo, Japan), and CD4^+^ T cells were purified by Phycoerythrin (PE)-conjugated anti-CD4 mAb, PE-conjugated CD8 mAb, and anti-PE Microbeads (Miltenyi Biotec). Cells were cultured in 96-well flat-bottom microtiter plates (5×10^4^ cells/well) in RPMI1640 containing 10% FCS, penicillin/streptomycin, and 2-mercaptoethanol (ME), and were stimulated with plate-coated anti-CD3 (500A2) (BD Biosciences) and anti-CD28 mAb (37.51) (BD Biosciences). [^3^H]Thymidine incorporation during the last 12 hours of the culture for 72 hours was evaluated using an automated β liquid scintillation counter. In addition, cells were labeled with carboxyfluorescein diacetate succinimidyl ester (CFSE), and dilution of CFSE was evaluated as cell proliferation after stimulation by flow cytometric analysis.

### ELISA

The amounts of mouse IL-2, IFN-γ, IL-4, IL-10, and IL-17 in culture supernatants, rheumatoid factor (RF) (IgM and IgG), anti-double strand (ds)DNA, anti-CII, and anti-nuclear Ab (ANA) (ALPHA DIAGNOSTIC INTERNATIONAL) of sera from the recipient MRL/*lpr* mice were detected by ELISA as previously described [14].

### Detection of apoptotic cells

To determine the apoptosis of T and B cells by repeated interactions with DCs, T and B cells were co-cultured for 8 hours three times with RANKL-stimulated MRL*/lpr* or MRL*+/+* BMDCs. Briefly, we transferred T cells into the other well, in which DCs had been stimulated, without interval three times for each 8 hours. After the third co-culture with DCs, apoptotic cells of CD4^+^, CD8^+^ T, DNT, and B cells were detected with flow cytometer using an Annexin V-FITC apoptosis detection kit (Bio Vision, Mountain View, CA). Purified anti-mouse TRAIL mAb (BioLegend, San Diego, CA, clone:N2B2) was used for inhibition of T cell apoptosis.

### Gene expression analysis

Apoptosis pathway-focused gene expression profiling analysis using real-time polymerase chain reaction (PCR) was tested with a PCR Primer array kit (SABiosciences Corporation, Frederick, MD, USA). In brief, total RNA was extracted with RNeasy kits (Qiagen Inc., Valencia, CA), and reverse transcribed. The synthesized cDNA was then applied to PCR-based SuperArray (SABiosciences) plates to detect expression of genes related to apoptosis using a PTC-200 DNA Engine Cycler (BioRad Kaboratories, Hercules, CA) with SYBR Premix Ex Taq (Takara, Kyoto, Japan).

### Real-time quantitative reverse transcription-polymerase chain reaction (RT-PCR)

Total RNA was extracted from the cultured CD4^+^ T cells of MRL/*lpr* mice using RNeasy kits (Qiagen), and reverse-transcribed. Transcript levels of TRAF3, caspase 8, Tnfrsf10b (TRAIL-R2), Bcl-2, and β-actin were observed using PTC-200 DNA Engine Cycler (BioRad) with SYBR Premix Ex Taq (Takara, Kyoto, Japan). The following primer sequences were used: for TRAF3, 5′-AGCAGCTGACTCTGGGACAT-3′ (forward) and 5′-CACCACACAGGGACAATCTG-3′ (reverse); for Tnfrsf10b (TRAIL-R2), 5′-ACTTGCTGAGAGCTGACTCTGTGG-3′ (forward) and 5′-AGCAGTGGCTGTGTTCACAAGG-3′ (reverse); for caspase 8, 5′-GAGATCCTGTGAATGGAACCTGGTA-3′ (forward) and 5′-CACGCCAGTCAGGATGCTAAGA-3′ (reverse); for Bcl-2, 5′-TTCGCAGCGATGTCCAGTCAGC-3′ (forward) and 5′-TGAAGAGTTCTTCCACCACCGT-3′ (reverse); and for β-actin, 5′-GTGGGCCGCTCTAGGCACCA-3′ (forward) and 5′-CGGTTGGCCTTAGGGTTCAGGGGGG-3′ (reverse). Relative mRNA abundance of each transcript was normalized against β-actin.

### *In vitro* knockdown of TRAIL gene in BMDCs

Small interfering RNA (siRNA) of TRAIL (Tnfs10) and a negative control (Stealth™ Select RNAi, Cat No:10620319, Invitrogen, Carlsbad, CA, USA), including three sequences as off-targets were used for analysis of *in vitro* knockdown of TRAIL gene in RANKL-stimulated DCs. Transfection of siRNA into DCs was performed with Lipofectamine™ RNAiMAX Reagent (Invitrogen).

### Statistical Analysis

Results are given as mean ± standard deviation (SD). Comparison was done using Student's *t* test. Differences were considered statistically significant for *P* values of <0.05.

## Supporting Information

Figure S1 **The effect of multiple transfers of activated** ***lpr*** **DCs on RA lesion and autoantibody production in MRL/** ***lpr*** **mice.** (A) RA lesions of recipient female mice treated with multiple transfers of DCs that were stimulated by different condition in vitro were compared. The histological score of the recipient mice (16 weeks of age) was evaluated at 12 weeks after repeated transfers. Data are shown as means ± SD (n = 5 per group respectively). (B) Autoantibody production of anti-dsDNA, anti-CII, and anti-nuclear Ab (ANA) of the sera from non-treated, stimulated +/+ DC-transferred, and stimulated *lpr* DC-transferred mice (16 weeks of age) was measured by ELISA. Data are shown as means ± SD (n = 5 per group respectively). (C) Autoantibody production of anti-dsDNA and anti-CII Abs of the sera from mice (16 weeks of age) transferred with RANKL, or RANKL and CII-stimulated *lpr* DCs was measured by ELISA. Data are shown as means ± SD (n = 5 per group respectively). *p<0.05.
(TIF)

Figure S2 **Apoptosis of T and B cells by multiple transfers of** ***lpr*** **DCs.** Apoptosis (annexin V^+^) of CD4^+^ (A), CD8^+^ (B) T, DNT (C), and B (CD19^+^) (D) cells of spleen and ILNs in the recipient mice was detected by flow cytometric analysis at 2 weeks after the multiple transfers. Data are shown as means ± SD (n = 3 per group respectively). *p<0.05, **p<0.005.
(TIF)

Figure S3 **Activation or maturation markers on T and B cells.** (A) T cell markers (CD44 and CD62L) of CD4^+^-gated cells of spleen and ILNs in the recipients (16 weeks of age) were analyzed by flow cytometry at 12 weeks after the multiple transfers. Results were representative of 5 mice per group. (B) B cell markers (CD27 and CD5) of CD19^+^-gated cells of spleen and ILNs in the recipients were analyzed by flow cytometry at 12 weeks after the multiple transfers. Results were representative of 5 mice per group.
(TIF)

Figure S4 **Survival of DCs in MRL/** ***lpr*** **mice.** BMDCs from MRL+/+ or MRL/lpr mice were stimulated with or without RANKL and CII, and then were labeled with CFSE. Those DCs were subcutaneously injected into MRL/*lpr* mice. At 2 weeks after the transfer, CFSE^+^CD11C^+^ DCs of ILNs (A) and spleen (B) were detected by flow cytometric analysis. Data are shown as means ± SD (n = 5 per group respectively). *p<0.05.
(TIF)

Figure S5 **Effect of multiple DC transfer on T cell proliferation in MRL+/+ mice.** At 12 weeks after multiple DC transfers into MRL+/+ mice, CFSE-labeled CD4^+^ T cells were stimulated with anti-CD3 (0.5 µg/ml) and anti-CD28 (10 µg/ml) mAbs for 72 hours. Dilution of CFSE in CD4^+^ T cells was evaluated as proliferative cells by flow cytometric analysis. Data are shown as means ± SD (n = 5 per group respectively).
(TIF)

Figure S6 **Effects of multiple DC transfer on thymic differentiation of T cell and T_reg_ differentiation.** (A) T cell phenotype (CD4 and CD8) in the thymus of the recipient mice was analyzed by flow cytometry at 12 weeks after the multiple DC transfer. Results were representative of 5 mice per group. (B) CD25^+^ Foxp3^+^ CD4^+^ T_reg_ cells in ILNs and spleen were detected by flow cytometric analysis. Results were representative of 5 mice per group.
(TIF)

Figure S7 **Efficiency of siRNA on TRAIL expression.** (A) TRAIL expression on *lpr* BMDCs treated with control or TRAIL siRNA (0, 10 and 50 nM) was detected by flow cytometric analysis. Results were representative of individual three experiments. (B) Relative expression of TRAIL to that of untreated DCs was shown.
(TIF)

Figure S8 **Effect of transfer of TRAIL siRNA-treated DCs on autoantibody production.** Autoantibodies such as anti-dsDNA and anti-CII Abs of the sera from mice (16 weeks of age) transferred with control and TRAIL siRNA-treated DCs were detected by ELISA. Data are shown as means ± SD (n = 5 per group respectively). *p<0.05.
(TIF)

## References

[1] Firestein GS (2003) Evolving concepts of rheumatoid arthritis. Nature 423: 356–361. 12748655 10.1038/nature01661

[2] McInnes IB, Schett G (2007) Cytokines in the pathogenesis of rheumatoid arthritis. Nat Rev Immunol 7: 429–442. 17525752 10.1038/nri2094

[3] Takayanagi H (2007) Osteoimmunology: shared mechanisms and crosstalk between the immune and bone systems. Nat Rev Immunol 7: 292–304. 17380158 10.1038/nri2062

[4] Merad M, Manz MG (2009) Dendritic cell homeostasis. Blood 113: 3418–3427. 19176316 10.1182/blood-2008-12-180646 PMC2668851

[5] Rescigno M, Martino M, Sutherland CL, Gold MR, Ricciardi-Castagnoli P (1998) Dendritic cell survival and maturation are regulated by different signaling pathways. J Exp Med 188: 2175–2180. 9841930 10.1084/jem.188.11.2175 PMC2212396

[6] Yamamoto M, Sato S, Hemmi H, Sanjo H, Uematsu S, et al (2002) Essential role for TIRAP in activation of the signalling cascade shared by TLR2 and TLR4. Nature 420: 324–329. 12447441 10.1038/nature01182

[7] Anderson DM, Maraskovsky E, Billingsley WL, Dougall WC, Tometsko ME, et al (1997) A homologue of the TNF receptor and its ligand enhance T-cell growth and dendritic-cell function. Nature 390: 175–179. 9367155 10.1038/36593

[8] Caux C, Massacrier C, Vanbervliet B, Dubois B, Kooten Van, et al (1994) Activation of human dendritic cells through CD40 cross-linking. J Exp Med 180: 1263–1272. 7523569 10.1084/jem.180.4.1263 PMC2191669

[9] Van Kooten C, Banchereau J (1997) Functions of CD40 on B cells, dendritic cells and other cells. Curr Opin Immunol 9: 330–337. 9203418 10.1016/s0952-7915(97)80078-7

[10] Van Gool SW, Vandenberghe P, de Boer M, Ceuppens JL (1996) CD80, CD86 and CD40 provide accessory signals in a multiple-step T-cell activation model. Immunol Rev 153: 47–83. 9010719 10.1111/j.1600-065x.1996.tb00920.x

[11] Banchereau J, Steinman RM (1998) Dendritic cells and the control of immunity. Nature 392: 245–252. 9521319 10.1038/32588

[12] van Duivenvoorde LM, van Mierlo GJ, Boonman ZF, Toes RE (2006) Dendritic cells: vehicles for tolerance induction and prevention of autoimmune diseases. Immunobiology 211: 627–632. 16920501 10.1016/j.imbio.2006.05.014

[13] Wenink MH, Han W, Toes RE, Radstake TR (2009) Dendritic cells and their potential implication in pathology and treatment of rheumatoid arthritis. Handb Exp Pharmacol 81–98. 19031022 10.1007/978-3-540-71029-5_4

[14] Izawa T, Ishimaru N, Moriyama K, Kohashi M, Arakaki R, et al (2007) Crosstalk between RANKL and Fas signaling in dendritic cells controls immune tolerance. Blood 110: 242–50. 17371940 10.1182/blood-2006-11-059980

[15] Wong BR, Josien R, Choi Y (1999) TRANCE is a TNF family member that regulates dendritic cell and osteoclast function. J Leukoc Biol 65: 715–24. 10380891 10.1002/jlb.65.6.715

[16] Yasuda H, Shima N, Nakagawa N, Yamaguchi K, Kinosaki M, et al (1998) Osteoclast differentiation factor is a ligand for osteoprotegerin/osteoclastogenesis-inhibitory factor and is identical to TRANCE/RANKL. Proc Natl Acad Sci U S A 95: 3597–3602. 9520411 10.1073/pnas.95.7.3597 PMC19881

[17] Lacey DL, Timms E, Tan HL, Kelley MJ, Dunstan CR, et al (1998) Osteoprotegerin ligand is a cytokine that regulates osteoclast differentiation and activation. Cell 93: 165–176. 9568710 10.1016/s0092-8674(00)81569-x

[18] Simonet WS, Lacey DL, Dunstan CR, Kelley M, Chang MS, et al (1997) Osteoprotegerin: a novel secreted protein involved in the regulation of bone density. Cell 89: 309–319. 9108485 10.1016/s0092-8674(00)80209-3

[19] Shalhoub V, Elliott G, Chiu L, Manoukian R, Kelley M, et al (2000) Characterization of osteoclast precursors in human blood. Br J Haematol 111: 501–512. 11122091 10.1046/j.1365-2141.2000.02379.x

[20] Kong YY, Yoshida H, Sarosi I, Tan HL, Timms E, et al (1999) OPGL is a key regulator of osteoclastogenesis, lymphocyte development and lymph-node organogenesis. Nature 397: 315–323. 9950424 10.1038/16852

[21] Fata JE, Kong YY, Li J, Sasaki T, Irie-Sasaki J, et al (2000) The osteoclast differentiation factor osteoprotegerin-ligand is essential for mammary gland development. Cell 103: 41–50. 11051546 10.1016/s0092-8674(00)00103-3

[22] Plows D, Kontogeorgos G, Kollias G (1999) Mice lacking mature T and B lymphocytes develop arthritic lesions after immunization with type II collagen. J Immunol 162: 1018–1023. 9916728

[23] Bonardelle D, Bobé P, Reynès M, Amouroux J, Tricottet V, et al (2001) Inflammatory arthropathy in MRL hematopoietic chimeras undergoing Fas mediated graft-versus-host syndrome. J Rheumatol 28: 956–961. 11361222

[24] Edwards CK 3rd, Zhou T, Zhang J, Baker TJ, De M, et al (1996) Inhibition of superantigen-induced proinflammatory cytokine production and inflammatory arthritis in MRL-lpr/lpr mice by a transcriptional inhibitor of TNF-alpha. J Immunol 157: 1758–1772. 8759766

[25] Watanabe-Fukunaga R, Brannan CI, Copeland NG, Jenkins NA, Nagata S (1992) Lymphoproliferation disorder in mice explained by defects in Fas antigen that mediates apoptosis. Nature 356: 314–317. 1372394 10.1038/356314a0

[26] Morse HC 3rd, Davidson WF, Yetter RA, Murphy ED, Roths JB, et al (1982) Abnormalities induced by the mutant gene Ipr: expansion of a unique lymphocyte subset. J Immunol 129: 2612–2615. 6815273

[27] Zhang XR, Zhang LY, Devadas S, Li L, Keegan AD, et al (2003) Reciprocal expression of TRAIL and CD95L in Th1 and Th2 cells: role of apoptosis in T helper subset differentiation. Cell Death Differ 10: 203–210. 12700648 10.1038/sj.cdd.4401138

[28] Fanger NA, Maliszewski CR, Schooley K, Griffith TS (1999) Human dendritic cells mediate cellular apoptosis via tumor necrosis factor-related apoptosis-inducing ligand (TRAIL). J Exp Med 190: 1155–1164. 10523613 10.1084/jem.190.8.1155 PMC2195665

[29] Jeremias I, Herr I, Boehler T, Debatin KM (1998) TRAIL/Apo-2-ligand-induced apoptosis in human T cells. Eur J Immunol 28: 143–152. 9485194 10.1002/(SICI)1521-4141(199801)28:01<143::AID-IMMU143>3.0.CO;2-3

[30] Sarkar S, Fox DA (2005) Dendritic cells in rheumatoid arthritis. Front Biosci 10: 656–665. 15569606 10.2741/1560

[31] Elliot MJ, Maini RN, Feldmann M, Long-Fox A, Charles P, et al (2008) Treatment of rheumatoid arthritis with chimeric monoclonal antibodies to tumor necrosis factor alpha. Arthritis Rheum 58: S92–S101. 18240199 10.1002/art.23362

[32] Menges M, Rossner S, Voigtlander C, Schindler H, Kukutsch NA, et al (2002) Repetitive injections of dendritic cells matured with tumor necrosis factor alpha induce antigen-specific protection of mice from autoimmunity. J Exp Med 195: 15–21. 11781361 10.1084/jem.20011341 PMC2196016

[33] Lopatin U, Yao X, Williams RK, Bleesing JJ, Dale JK, et al (2001) Increases in circulating and lymphoid tissue interleukin-10 in autoimmune lymphoproliferative syndrome are associated with disease expression. Blood 97: 3161–3170. 11342444 10.1182/blood.v97.10.3161

[34] Prud'homme GJ, Kono DH, Theofilopoulos AN (1995) Quantitative polymerase chain reaction analysis reveals marked overexpression of interleukin-1 beta, interleukin-1 and interferon-gamma mRNA in the lymph nodes of lupus-prone mice. Mol Immunol 32: 495–503. 7783752 10.1016/0161-5890(95)00024-9

[35] Llorente L, Zou W, Levy Y, Richaud-Patin Y, Wijdenes J, et al (1995) Role of interleukin 10 in the B lymphocyte hyperactivity and autoantibody production of human systemic lupus erythematosus. J Exp Med 181: 839–44. 7869046 10.1084/jem.181.3.839 PMC2191898

[36] Houssiau FA, Lefebvre C, Vanden Berghe M, Lambert M, Devogelaer JP, et al (1995) Serum interleukin 10 titers in systemic lupus erythematosus reflect disease activity. Lupus 4: 393–395. 8563734 10.1177/096120339500400510

[37] Wu X, Pan G, Mckenna MA, Zayzafoon M, Xiong WC, et al (2005) RANKL regulates Fas expression and Fas-mediated apoptosis in osteoclasts. J Bone Miner Res 20: 107–116. 15619676 10.1359/JBMR.041022

[38] Pan G, Ni J, Wei YF, Yu G, Gentz R, et al (1997) An antagonist decoy receptor and a death domain-containing receptor for TRAIL. Science 277: 815–818. 9242610 10.1126/science.277.5327.815

[39] Schneider P, Bodmer JL, Thome M, Hofmann K, Holler N, et al (1997) Characterization of two receptors for TRAIL. FEBS Lett 416: 329–334. 9373179 10.1016/s0014-5793(97)01231-3

[40] Sheikh MS, Huang Y, Fernandez-Salas EA, El-Deiry WS, Friess H, et al (1999) The antiapoptotic decoy receptor TRID/TRAIL-R3 is a p53-regulated DNA damage-inducible gene that is overexpressed in primary tumors of the gastrointestinal tract. Oncogene 18: 4153–4159. 10435597 10.1038/sj.onc.1202763

[41] Sheridan JP, Marsters SA, Pitti RM, Gurney A, Skubatch M, et al (1997) Control of TRAIL-induced apoptosis by a family of signaling and decoy receptors. Science 277: 818–821. 9242611 10.1126/science.277.5327.818

[42] Sprick MR, Rieser E, Stahl H, Grosse-Wilde A, Weigand MA, et al (2002) Caspase-10 is recruited to and activated at the native TRAIL and CD95 death-inducing signalling complexes in a FADD-dependent manner but can not functionally substitute caspase-8. Embo J 21: 4520–4530. 12198154 10.1093/emboj/cdf441 PMC126181

[43] Bodmer JL, Holler N, Reynard S, Vinciguerra P, Schneider P, et al (2000) TRAIL receptor-2 signals apoptosis through FADD and caspase-8. Nat Cell Biol 2: 241–243. 10783243 10.1038/35008667

[44] Hersey P, Zhang XD (2001) How melanoma cells evade trail-induced apoptosis. Nat Rev Cancer 1: 142–150. 11905805 10.1038/35101078

[45] Liu Z, Xu X, Hsu HC, Tousson A, Yang PA, et al (2003) CII-DC-AdTRAIL cell gene therapy inhibits infiltration of CII-reactive T cells and CII-induced arthritis. J Clin Invest 112: 1332–1341. 14597760 10.1172/JCI19209 PMC228459

[46] Krammer PH, Arnold R, Lavrik IN (2007) Life and death in peripheral T cells. Nat Rev Immunol 7: 532–542. 17589543 10.1038/nri2115

[47] Russell JH, Ley TJ (2002) Lymphocyte-mediated cytotoxicity. Annu Rev Immunol 20: 323–370. 11861606 10.1146/annurev.immunol.20.100201.131730

[48] Lamhamedi-Cherradi SE, Zheng SJ, Maguschak KA, Peschon J, Chen YH (2003) Defective thymocyte apoptosis and accelerated autoimmune diseases in TRAIL-/- mice. Nat Immunol 4: 255–260. 12577054 10.1038/ni894

[49] Inaba K, Inaba M, Romani N, Aya H, Deguchi M, et al (1992) Generation of large numbers of dendritic cells from mouse bone marrow cultures supplemented with granulocyte/macrophage colony-stimulating factor. J Exp Med 176: 1693–1702. 1460426 10.1084/jem.176.6.1693 PMC2119469

[50] Andrews BS, Eisenberg RA, Theofilopoulos AN, Izui S, Wilson CB, et al (1978) Spontaneous murine lupus-like syndromes. Clinical and immunopathological manifestations in several strains. J Exp Med 148: 1198–1215. 309911 10.1084/jem.148.5.1198 PMC2185049

[51] Hang L, Theofilopoulos AN, Dixon FJ (1982) A spontaneous rheumatoid arthritis-like disease in MRL/l mice. J Exp Med 155: 1690–1701. 7077223 10.1084/jem.155.6.1690 PMC2186697

[52] O'Sullivan FX, Fassbender HG, Gay S, Koopman WJ (1985) Etiopathogenesis of the rheumatoid arthritis-like disease in MRL/l mice. I. The histomorphologic basis of joint destruction. Arthritis Rheum 28 5: 529–536. 4004962 10.1002/art.1780280511

[53] Tarkowski A, Jonsson R, Holmdahl R, Klareskog L (1987) Immunohistochemical characterization of synovial cells in arthritic MRL-lpr/lpr mice. Arthritis Rheum 30: 75–82. 3814199 10.1002/art.1780300110

