# Supplementary figures and images for: Fas-Independent T-Cell Apoptosis by Dendritic Cells Controls Autoimmune Arthritis in MRL/lpr Mice
Source: PLoS One. 2012 Dec 12;7(12):e48798. doi: 10.1371/journal.pone.0048798 (PMC3523790; doi:10.1371/journal.pone.0048798)

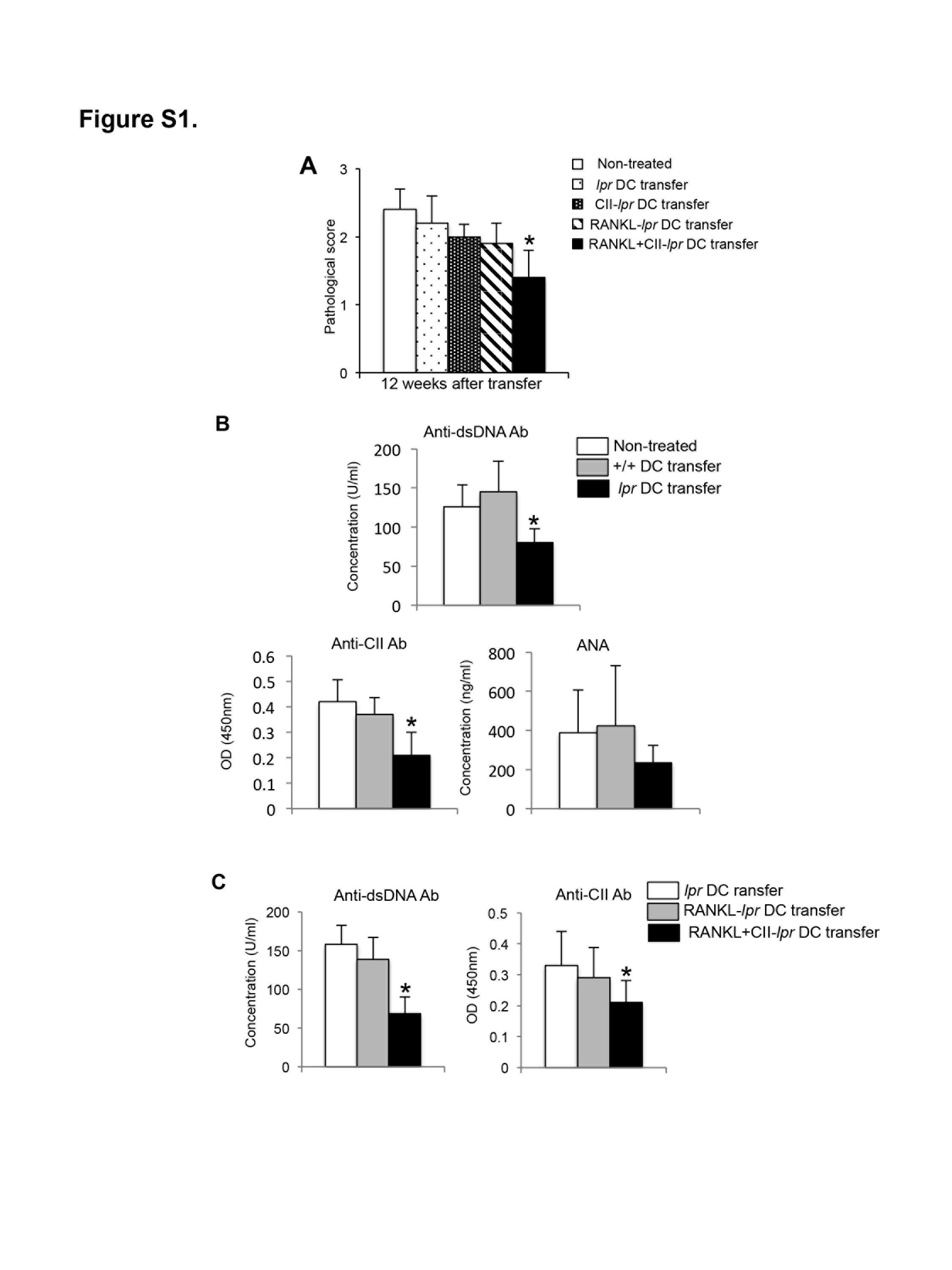

Supplement: Figure S1 — The effect of multiple transfers of activated lpr DCs on RA lesion and autoantibody production in MRL/ lpr mice. (A) RA lesions of recipient female mice treated with multiple transfers of DCs that were stimulated by different condition in vitro were compared. The histological score of the recipient mice (16 weeks of age) was evaluated at 12 weeks after repeated transfers. Data are shown as means ± SD (n = 5 per group respectively). (B) Autoantibody production of anti-dsDNA, anti-CII, and anti-nuclear Ab (ANA) of the sera from non-treated, stimulated +/+ DC-transferred, and stimulated lpr DC-transferred mice (16 weeks of age) was measured by ELISA. Data are shown as means ± SD (n = 5 per group respectively). (C) Autoantibody production of anti-dsDNA and anti-CII Abs of the sera from mice (16 weeks of age) transferred with RANKL, or RANKL and CII-stimulated lpr DCs was measured by ELISA. Data are shown as means ± SD (n = 5 per group respectively). *p<0.05. (TIF) [file pone.0048798.s001.tif]

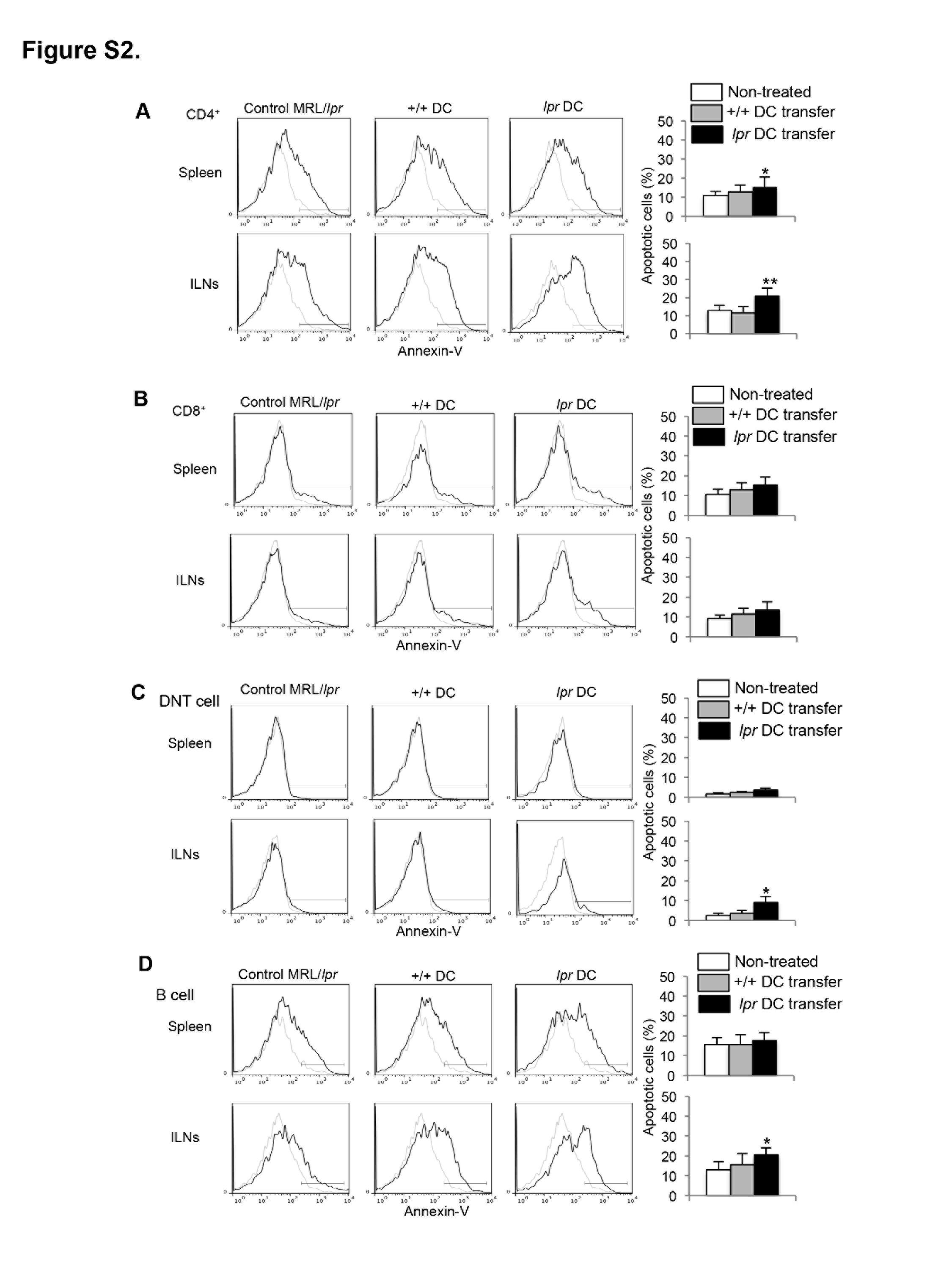

Supplement: Figure S2 — Apoptosis of T and B cells by multiple transfers of lpr DCs. Apoptosis (annexin V+) of CD4+ (A), CD8+ (B) T, DNT (C), and B (CD19+) (D) cells of spleen and ILNs in the recipient mice was detected by flow cytometric analysis at 2 weeks after the multiple transfers. Data are shown as means ± SD (n = 3 per group respectively). *p<0.05, **p<0.005. (TIF) [file pone.0048798.s002.tif]

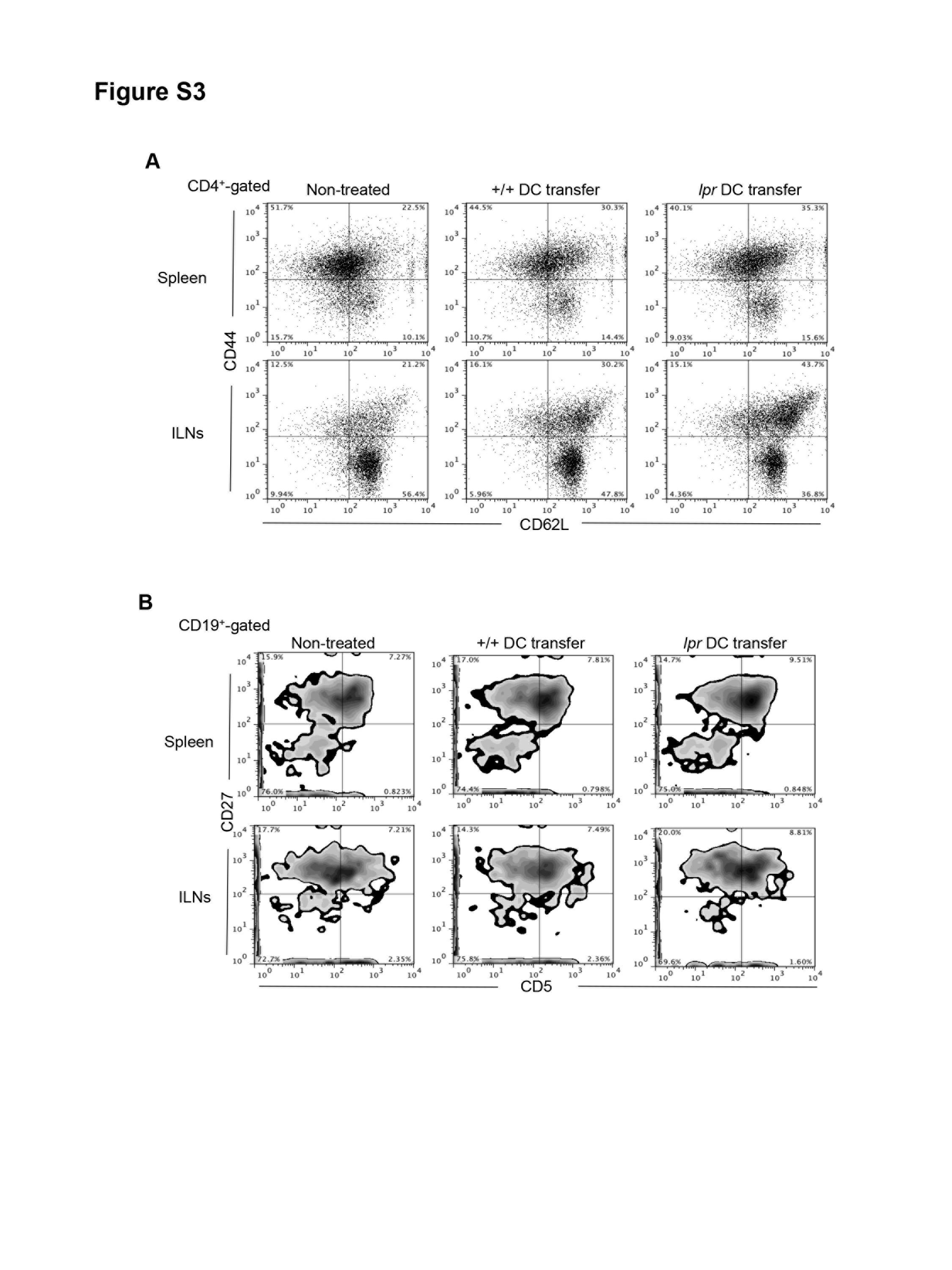

Supplement: Figure S3 — Activation or maturation markers on T and B cells. (A) T cell markers (CD44 and CD62L) of CD4+-gated cells of spleen and ILNs in the recipients (16 weeks of age) were analyzed by flow cytometry at 12 weeks after the multiple transfers. Results were representative of 5 mice per group. (B) B cell markers (CD27 and CD5) of CD19+-gated cells of spleen and ILNs in the recipients were analyzed by flow cytometry at 12 weeks after the multiple transfers. Results were representative of 5 mice per group. (TIF) [file pone.0048798.s003.tif]

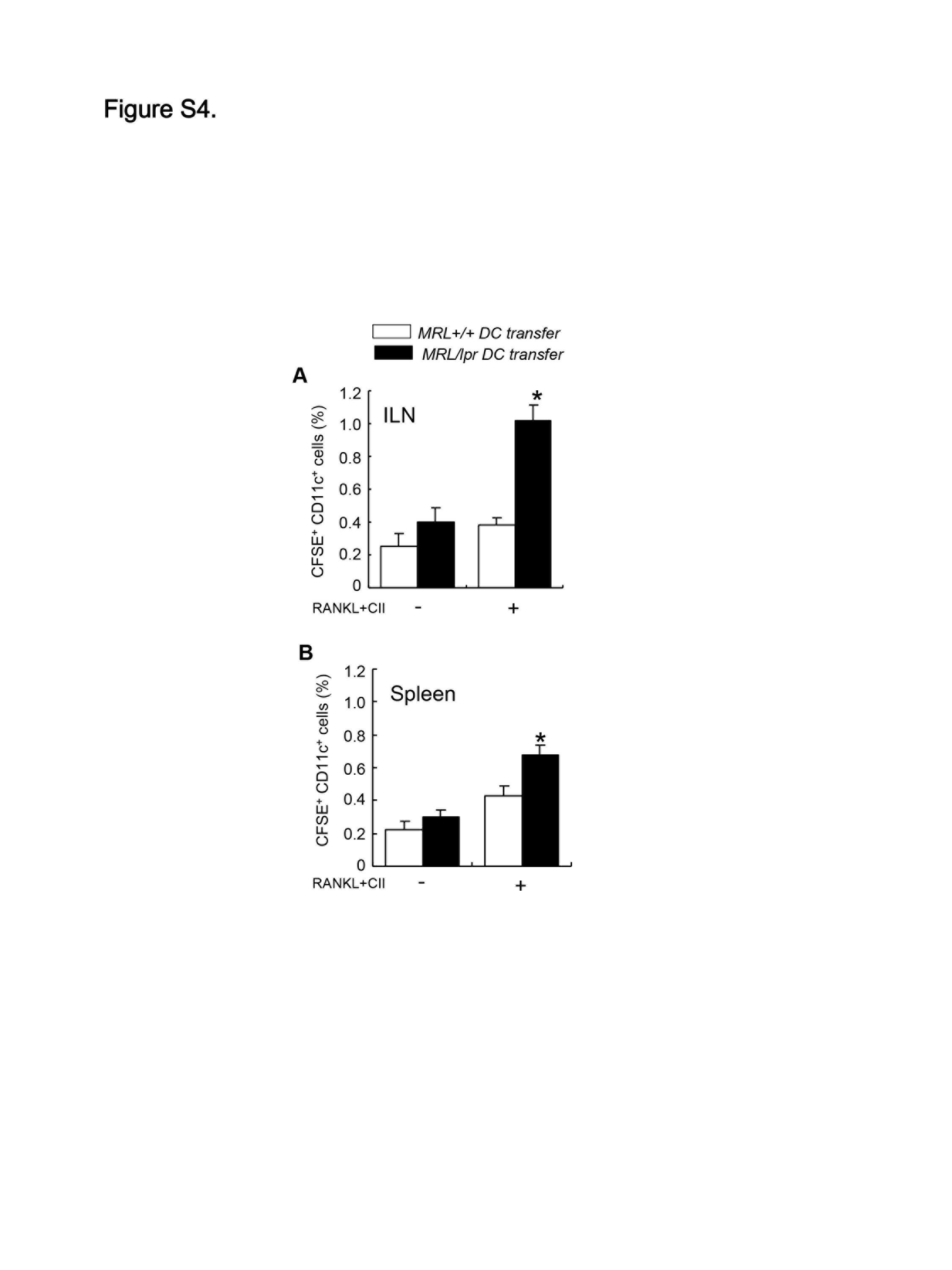

Supplement: Figure S4 — Survival of DCs in MRL/ lpr mice. BMDCs from MRL+/+ or MRL/lpr mice were stimulated with or without RANKL and CII, and then were labeled with CFSE. Those DCs were subcutaneously injected into MRL/lpr mice. At 2 weeks after the transfer, CFSE+CD11C+ DCs of ILNs (A) and spleen (B) were detected by flow cytometric analysis. Data are shown as means ± SD (n = 5 per group respectively). *p<0.05. (TIF) [file pone.0048798.s004.tif]

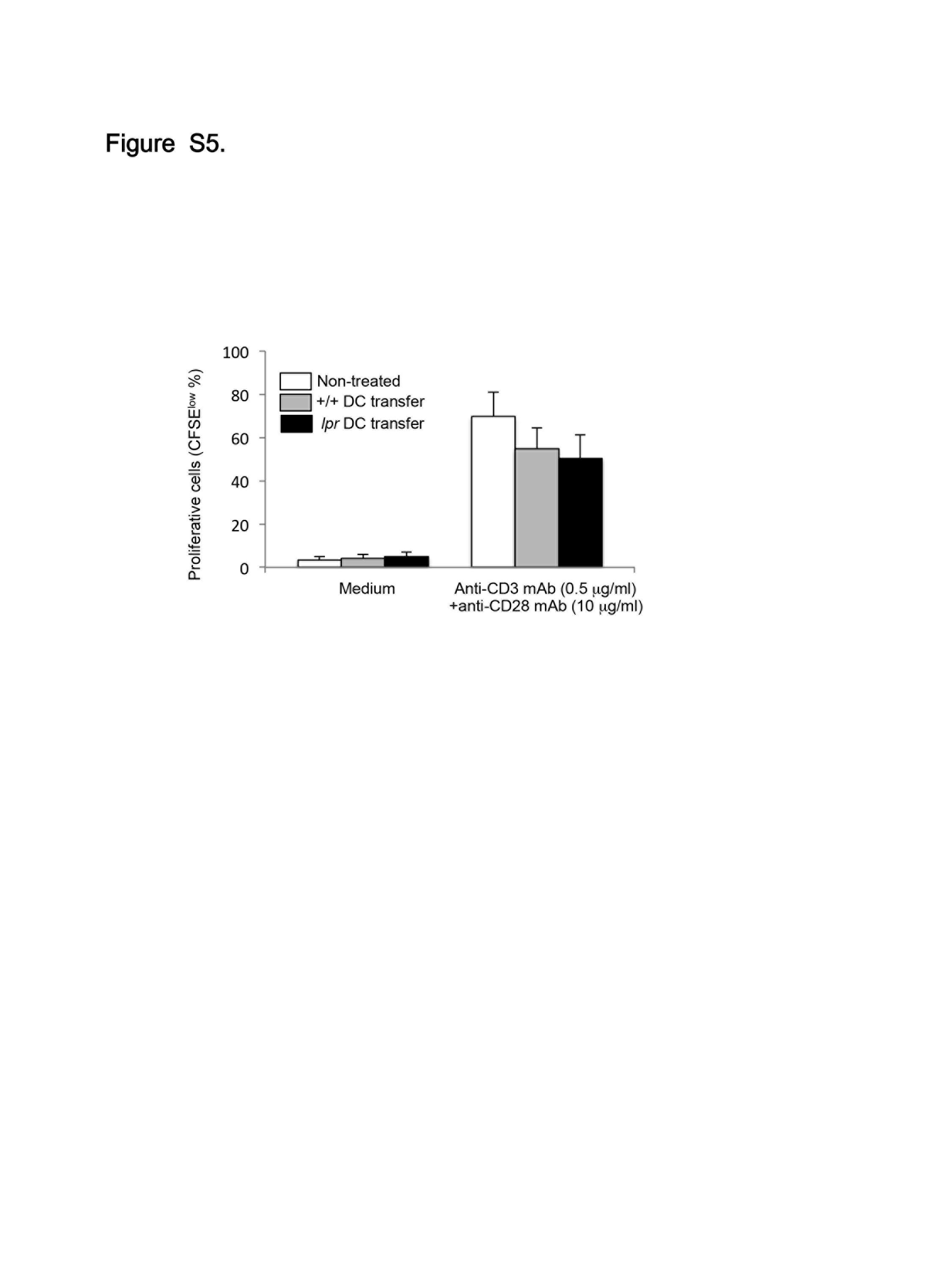

Supplement: Figure S5 — Effect of multiple DC transfer on T cell proliferation in MRL+/+ mice. At 12 weeks after multiple DC transfers into MRL+/+ mice, CFSE-labeled CD4+ T cells were stimulated with anti-CD3 (0.5 µg/ml) and anti-CD28 (10 µg/ml) mAbs for 72 hours. Dilution of CFSE in CD4+ T cells was evaluated as proliferative cells by flow cytometric analysis. Data are shown as means ± SD (n = 5 per group respectively). (TIF) [file pone.0048798.s005.tif]

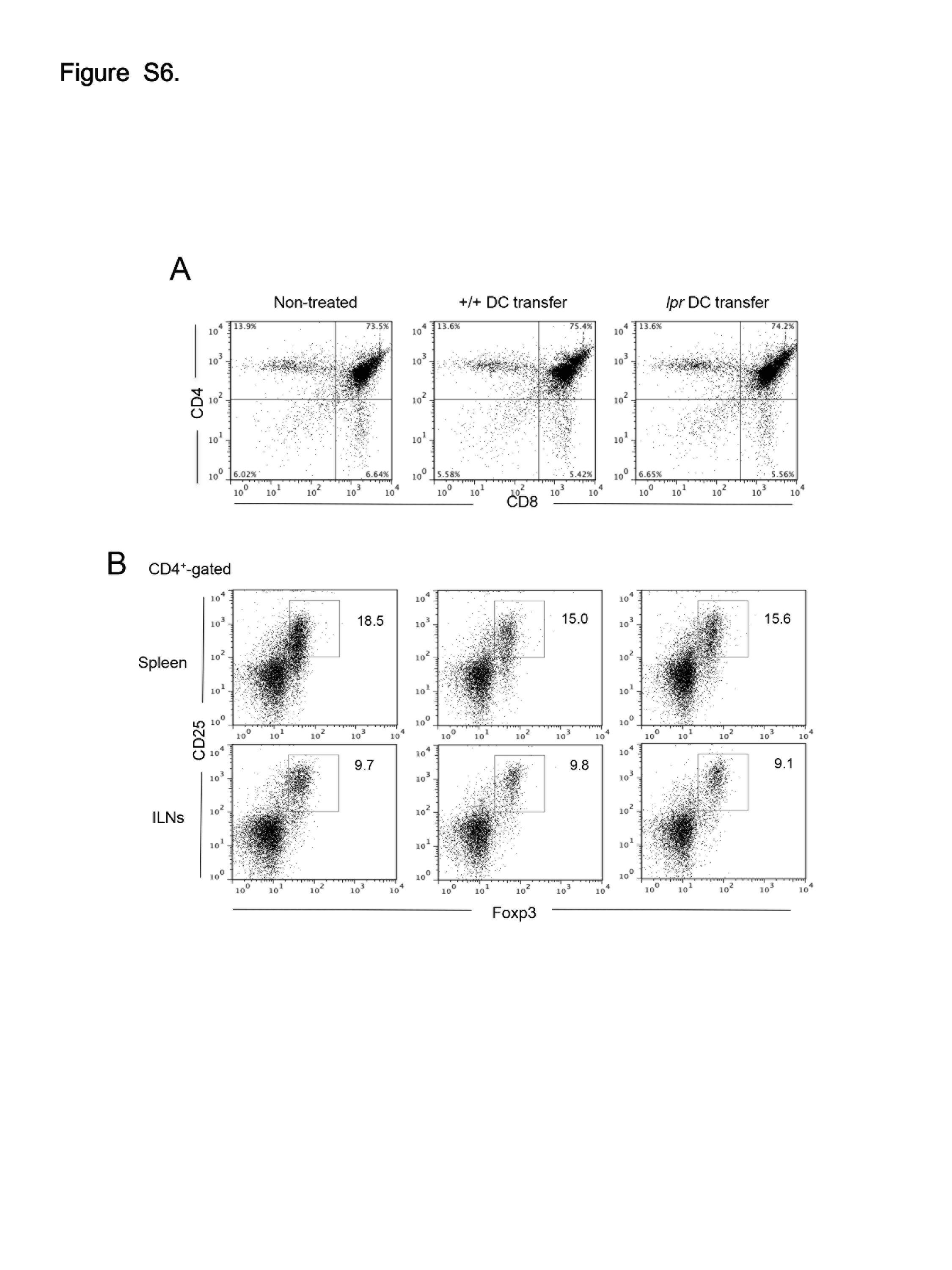

Supplement: Figure S6 — Effects of multiple DC transfer on thymic differentiation of T cell and Treg differentiation. (A) T cell phenotype (CD4 and CD8) in the thymus of the recipient mice was analyzed by flow cytometry at 12 weeks after the multiple DC transfer. Results were representative of 5 mice per group. (B) CD25+ Foxp3+ CD4+ Treg cells in ILNs and spleen were detected by flow cytometric analysis. Results were representative of 5 mice per group. (TIF) [file pone.0048798.s006.tif]

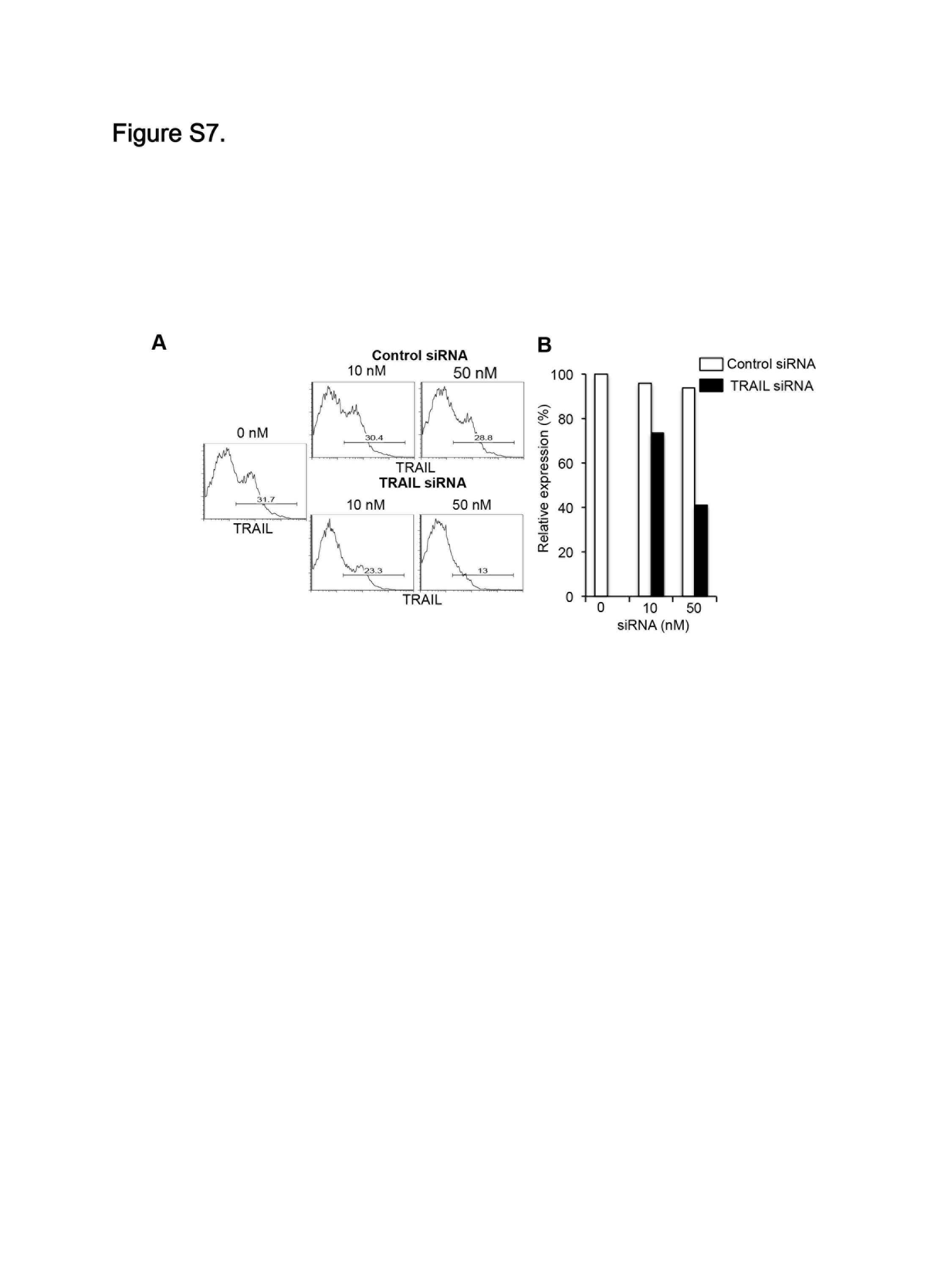

Supplement: Figure S7 — Efficiency of siRNA on TRAIL expression. (A) TRAIL expression on lpr BMDCs treated with control or TRAIL siRNA (0, 10 and 50 nM) was detected by flow cytometric analysis. Results were representative of individual three experiments. (B) Relative expression of TRAIL to that of untreated DCs was shown. (TIF) [file pone.0048798.s007.tif]

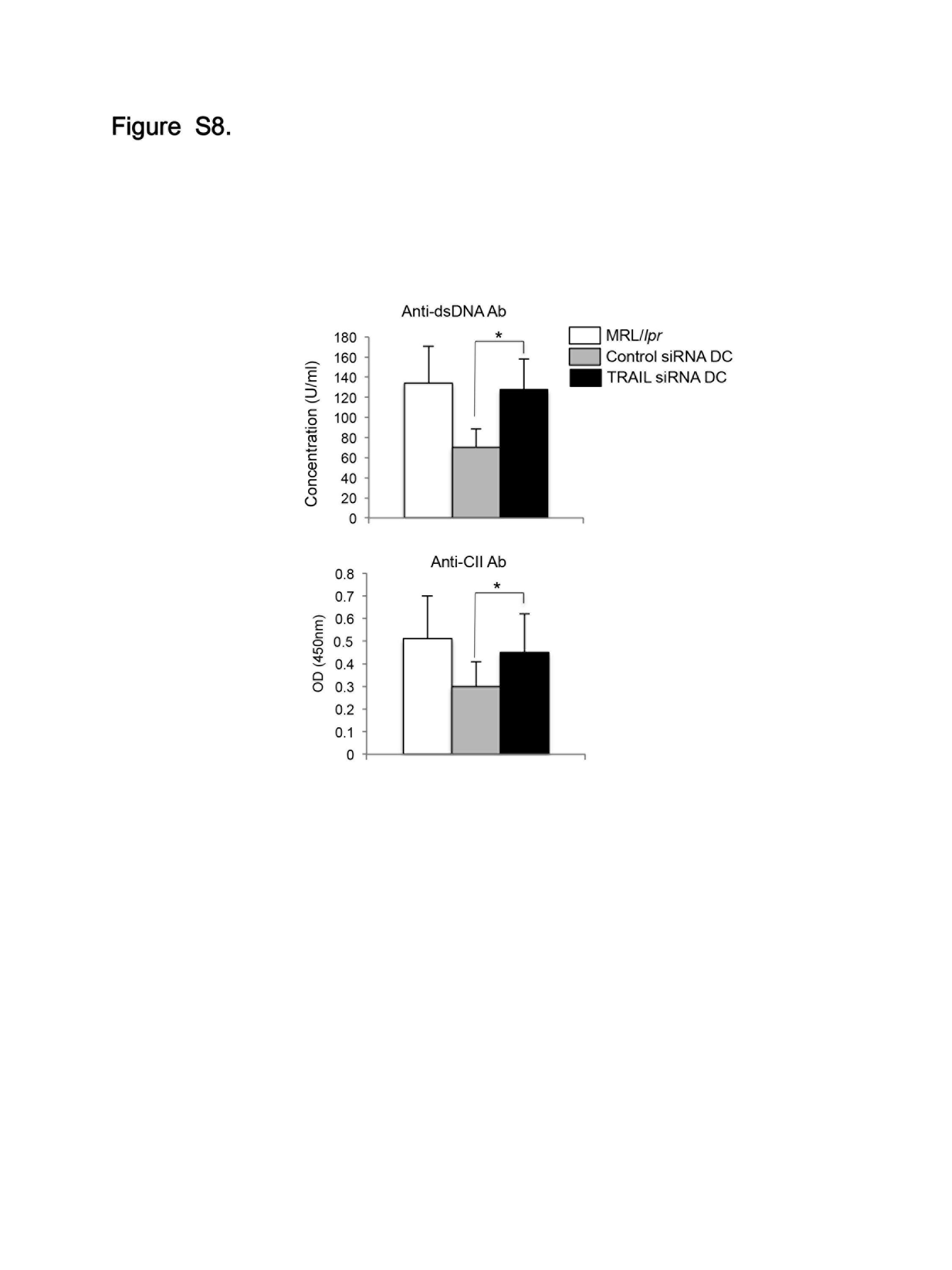

Supplement: Figure S8 — Effect of transfer of TRAIL siRNA-treated DCs on autoantibody production. Autoantibodies such as anti-dsDNA and anti-CII Abs of the sera from mice (16 weeks of age) transferred with control and TRAIL siRNA-treated DCs were detected by ELISA. Data are shown as means ± SD (n = 5 per group respectively). *p<0.05. (TIF) [file pone.0048798.s008.tif]
